# Hybrid Health IT and Telehealth–Delivered Behavioral Weight Loss Services for Primary Care Patients With Cardiovascular Risk Factors: Intervention Component Design and Pragmatic Randomized Feasibility Trial

**DOI:** 10.2196/58722

**Published:** 2025-10-22

**Authors:** Ronald T Ackermann, Kenzie A Cameron, David T Liss, Nancy Dolan, Cassandra Aikman, Amy R Carson, Sterling A Harris, Kathryn Doyle, Andrew J Cooper, Brian Hitsman

**Affiliations:** 1 Department of Medicine, Feinberg School of Medicine, Northwestern University, Chicago, IL, United States; 2 Northwestern Medicine, Chicago, IL, United States; 3 Institute for Public Health and Medicine, Feinberg School of Medicine, Northwestern University, 750 N Lake Shore Drive, Suite 609, Chicago, IL, 60611, United States, 1 3125036400; 4 Department of Preventive Medicine, Feinberg School of Medicine, Northwestern University, Chicago, IL, United States; 5 Fitness Formula Clubs, Chicago, IL, United States

**Keywords:** mHealth, mobile health, mobile application, app, application, obesity, overweight, obese, weight loss, cardiovascular, cardiovascular risk factor, risk factor, cardiovascular disease, CVD, cardiovascular illness, diabetes, primary prevention, behavioral intervention, lifestyle intervention, implementation, health care delivery, primary care, feasibility

## Abstract

**Background:**

Intensive lifestyle interventions (ILI) improve weight loss and cardiovascular risk factors, but health care systems face challenges in implementing them. We engaged stakeholders to cocreate and evaluate primary care implementation strategies for ILI components.

**Objective:**

This study aimed to describe the design of intervention components and implementation strategies and to evaluate the feasibility of pragmatic trial enrollment and randomization procedures, as well as the acceptability and preliminary effectiveness of the interventions.

**Methods:**

The study setting was a single, urban primary care office. Patients with a BMI ≥27 kg/m² and ≥1 cardiovascular risk factor were sent a single electronic health record (EHR) message between December 2019 and January 2020 offering services to support a weight loss goal of 10 pounds in 10 weeks. All patients who affirmed interest were pragmatically enrolled in a trial offering basic lifestyle support (BLS), which provided a scale that transmits weight data to the EHR using cellular networks, a coupon to enroll in lifestyle coaching resources through a partnering fitness organization, and periodic EHR messages encouraging use of those resources. About half (n=42) of participants were randomized by an EHR algorithm to also receive customized lifestyle support (CLS), including weekly email messages adaptive to weight loss progress and telephonic coaching by a nurse for those facing challenges. Interventions and assessments spanned from January to July 2020, with disruption by the COVID-19 pandemic. Weight data were collected from administrative sources. Qualitative analysis of stakeholder recommendations and patient interviews assessed intervention acceptability, appropriateness, and sustainability.

**Results:**

Over 6 weeks, 426 patients were sent the EHR invitation message, and 80 (18.8%) patients affirmed interest in the weight loss goal and were enrolled. Overall, 48 of 80 (60%) trial participants lost weight at 6 months; 12 (15%) exhibited weight loss ≥5%, with no significant difference between CLS and BLS arms (*P*=.85). During the 12 weeks of adaptive MyChart (Epic Systems) messaging, 18 (43%) CLS patients and 8 (21%) BLS patients performed daily self-weighing (*P*=.06), and 22 (52%) CLS patients and 14 (37%) BLS patients enrolled in referral-based lifestyle resources (*P*=.18).

**Conclusions:**

Pragmatic enrollment, randomization, and data collection procedures proved feasible, and interventions showed preliminary effectiveness warranting further study in a larger trial.

## Introduction

Moderate-intensity physical activity and just 10 to 15 pounds of weight loss can improve blood glucose, blood pressure, and cholesterol and reduce the need for medications to control those cardiovascular risk factors [1-6]. Achieving and maintaining weight loss is difficult, particularly if the environment surrounding individuals is not supportive of healthy eating and physical activity [7]. Adults are more likely to achieve weight loss and activity goals when supported by behavioral interventions involving recurring contact with a behavioral coach over months to years [1,2]. Such “intensive lifestyle interventions” (ILIs) improve health-related quality of life, reduce work absence, and lower future health care usage and costs [8,9].

US health care systems are a natural partner for ILI implementation because 5 in 6 adults complete office visits each year [10], during which cardiovascular risk factors such as body weight, blood pressure, glucose, and cholesterol are routinely assessed. Health care systems also have incentives to intervene before cardiovascular risk factors cause symptoms, functional impairments, or costly health complications [9]. However, health care systems face challenges in implementing ILI [11,12], which require dedicated personnel, space, supplies, and technologies that generally do not align well with the existing structure, workforce, and routines of health care delivery.

Past research has investigated strategies to simplify the role of health care systems in ILI delivery. Examples include reducing the frequency or duration of behavioral coaching contacts or shifting the source of coaching to outside health care settings, such as via wellness professionals employed by community organizations or distance coaching delivered using a computer or smartphone [13-17]. These strategies have lower effectiveness than more intensive interventions studied in randomized controlled trials [18], but also cost less and reach more people [8,19,20]. Despite increasing numbers of ILI programs nationally, clinicians refer very few patients to ILIs, and most people who may benefit remain unaware [19]. There is an immense need for research that identifies feasible and acceptable strategies for health care systems to implement ILIs.

One approach used to adapt and improve the implementation of multicomponent behavioral interventions without eroding their fidelity is to (1) distill them to their core components and (2) devise simpler implementation strategies for each component that improve their feasibility and acceptability within health care system delivery settings [21,22]. When there are multiple feasible implementation strategies for each core component, this approach also offers opportunities for health care systems to customize intervention delivery based on individual patient preferences (eg, letting them choose between formats) or their responsiveness (eg, offering “option A” but stepping up to offer more intensive “option B” if patients do not engage or improve). This approach holds promise for expanding the reach of high-fidelity intervention delivery to many more patients, by offering less intensive ways to get started for those who are initially unable or unwilling to enroll when referred to a full-scale program offered in a single format or location, as well as by better aligning opportunities for engagement in each component with different forms of motivation and ability. Extensive previous research over the past 2 decades has helped to define the core components of ILIs to include support for: an initial weight loss goal of at least 5 to 10 kg (11 to 22 pounds) at a pace of about 0.5 to 2 pounds per week [1]; resources and training for individuals to self-monitor dietary, activity, and weight changes [23]; and longitudinal coaching (typically with contact every 1 to 4 weeks). Coaching serves to create accountability [24], develop self-regulation and problem-solving skills [25,26], suggest refinements to behavioral strategies, and build self-efficacy and mastery over behaviors [23]; coaching can be effective when offered face-to-face or via a technology platform, with individual participants or groups [1].

Over 3 years, we engaged with health care system and fitness industry leaders, managers, and service professionals to cocreate and evaluate the feasibility of an array of practical implementation strategies for health care systems to support ILI core components. To our knowledge, this is the first such effort in which all intervention components are embedded within or coordinated directly by an electronic health record (EHR) management system. This paper aims to describe the collaborative approach used for the creation of the ILI implementation strategies and to report preliminary data for the feasibility, acceptability, and potential effectiveness of this approach.

## Methods

### Research Approaches

Our research involved both stakeholder-engaged “cocreation” of implementation strategies using collaborative design principles and a small, parallel, 2-arm, pilot trial with 1:1 individual treatment assignment to demonstrate feasibility and acceptability of the strategies in a single general internal medicine practice setting in downtown Chicago, Illinois, United States. The trial involved pragmatic recruitment, enrollment, and data collection strategies [27]. Eligibility determination and intervention allocation were automated within the EHR; trial enrollment proceeded with an Institutional Review Board (IRB)-approved waiver of written informed consent, and most outcomes were ascertained from administrative data systems. Each of these approaches minimized contact between study patients and the research team and enabled study patients and the interventions they received to approximate “real-world” conditions. Eligible patients were enrolled from December 2019 to January 2020; data were collected through January 2021. The experimental services were offered to patients beginning in January 2020, and access to some services (described below in the “Patient Engagement in Intervention Components” subheading) was interrupted by the COVID-19 pandemic stay-at-home order in March 2020. All study procedures, including pragmatic enrollment and randomization under a waiver of written informed consent for research, were approved by the Northwestern University IRB, and the pilot trial was registered and posted on June 25, 2019, at ClinicalTrials.gov (NCT03998046). This paper was structured to comply with the CONSORT-EHEALTH (Consolidated Standards Of Reporting Trials for Electronic Health) Interventions reporting guidelines [28].

### Stakeholder Engagement in the Research

From September 2016 to December 2019, we engaged stakeholders to consider research evidence for ILI core components [1,2,23] and cocreate implementation strategies for sustainable delivery of each core component in busy primary care practice settings. Recurring meetings of a “design team” included behavioral and social scientists, health care system nursing supervisors, a practicing primary care physician, 2 wellness program leaders from the partnering fitness club network, and health care system employees who develop and manage new tools and applications within the EHR system. Individual meetings were held with additional stakeholders, including health care system leaders, medical group and practice leaders, and the CEO and wellness program leaders from a regional fitness club network. Research staff members organized meetings, kept detailed notes, verified recommendations and action steps with participants, and integrated ideas into an array of design themes and concepts that informed intervention prototypes, pretests, and refinements [11].

### Eligibility Criteria and Recruitment of Feasibility Trial Participants

A clinical data manager used EHR reporting tools to generate lists of eligible patients. Inclusion criteria were: age 18‐75 years; ≥1 log-in within the past 6 months to MyChart (ie, a secure EHR access portal for patients to view their chart and exchange information with care team members; Epic Systems); completion of ≥1 office visit in the past 6 months at the participating primary care practice site; measured weight and height at the most recent office visit indicating BMI ≥27.0 kg/m^2^; and ≥1 cardiometabolic risk factor (high blood pressure, abnormal blood cholesterol, prediabetes, or type 2 diabetes). Patients were ineligible if their last blood pressure was ≥180/105 mm Hg or their last hemoglobin A1c was ≥10.9%. Patients were also ineligible if they received cancer treatment in the past 2 years, were hospitalized in the past 3 months, were pregnant, or had a personal history of an eating disorder or serious mental illness.

The data manager used EHR messaging tools to send each physician a list of their own eligible patients, asking them to reply within 1 week if they wished to remove patients for whom they believed offering support for lifestyle change would be inappropriate. After 1 week, the data manager used the EHR’s batch patient messaging tool to transmit a standardized outreach “goal setting” message to each patient remaining on the list. The message encouraged healthy eating and physical activity changes with a weight loss goal, and it offered free-of-charge access to technologies and coaching support to help recipients achieve the goal. The message also prompted patients to click an embedded link if they wished to adopt a weight loss goal and receive support. Because limited resources were available to support intervention costs during feasibility testing, we sent MyChart messages to only 100‐200 patients every 2 weeks until the target sample size of 80 patients had affirmed interest in receiving the intervention services. This sample size was believed sufficient to demonstrate the feasibility and fidelity of pragmatic randomization and data collection procedures, to test the deployment of all variations of the patient messages, and to collect robust qualitative data about intervention perceptions. All 80 patients were included in the pragmatic trial evaluation; all received a bundle of basic lifestyle support (BLS) services (below in the “Procedures for Designing Intervention Components and Implementation Strategies” subheading), and about half (n=42) of patients were randomly assigned by a “silent” EHR algorithm to also receive additional, more customized lifestyle support (CLS) services (below in the “Procedures for Designing Intervention Components and Implementation Strategies” subheading). Both interventions are inherently unblinded.

After the trial enrollment target was reached, study patients were randomly selected for recruitment to complete semistructured telephone interviews to assess perceptions toward the intervention components. A research assistant emailed and then phoned 30 study patients before a targeted sample size of 15 interviews had been completed.

### Procedures for Designing Intervention Components and Implementation Strategies

Design goals were to cocreate ILI intervention components and implementation strategies that are evidence-based [1,2] and perceived by stakeholders as technically feasible, acceptable, and financially sustainable [29]. This approach followed collaborative design principles [11] and produced ILI implementation strategies for support of weight loss goal setting, self-weighing, and recurring coaching support. Stakeholders advised using the EHR to identify eligible patients, to message them to encourage a weight loss goal, inspire accountability, and increase engagement in coaching support services, as well as to monitor progress metrics as a means to offer adaptive, higher intensity services to patients facing challenges with automated support alone (Table S1 in Multimedia Appendix 1). Stakeholders cautioned that encouragement of a weight loss goal in a MyChart message could be perceived as inappropriate by some patients; they advised framing the goal and offering resources instead as a means to improve cardiovascular risk factor control. They also advised recommending a relatively simple initial goal of “about 10 pounds over 10 weeks,” believing this would be acceptable, appropriate, and achievable for most patients. This initial goal aligns with the large evidence base for effective ILIs, which typically support an initial weight loss goal of at least 5 kg (11 pounds) at a pace of about 0.5 to 2 pounds per week [1]. Stakeholders advised exploring the feasibility of receiving data from a wireless scale and analyzing that data directly within the EHR as a means to inform more personalized feedback about self-weighing and to identify and target patients with more adaptive forms of intervention support. They advised providing all patients with free-of-charge access to coaching services offered by a partnering fitness organization and assigning a practice nurse to provide “step-up” telephonic coaching to patients who do not make progress toward their weight goal. They emphasized that we should evaluate the quality of all new resources, whether patients perceive them as appropriate, and whether their costs are sustainable.

The resulting intervention components, which are described in detail in Multimedia Appendix 1, were all deployed or coordinated by the EHR. Interventions commenced when a patient received the introductory MyChart goal-setting message (above in the “Eligibility Criteria and Recruitment of Feasibility Trial Participants” subheading) and clicked the link to affirm interest in a weight loss goal. All 80 patients received, free of charge, a bundle of BLS services. Specifically, they were shipped an electronic weight scale (e-Scale), which had been programmed to transmit weight data back to their electronic chart using cellular networks, at no cost to the patient. Each patient received a follow-up MyChart message with simple instructions for using the scale to weigh themselves daily, as well as a “coupon code” to enroll free of charge in longitudinal lifestyle coaching and support resources offered by a regional fitness organization partner. Patients could choose to access the referral services in 2 formats: face-to-face access to a dietitian and fitness coach at 11 facility locations or fully remote access to the same professionals via video chat with a smartphone, tablet, or personal computer. The face-to-face option provided full health club facility access, and the remote delivery option provided a suite of healthy food preparation and physical activity tips, tracking tools, and videos. Each patient also received instructions for messaging a dedicated nurse-coach at the primary care practice if they had problems using the support services or if they wanted more personalized support for reaching dietary, physical activity, or weight loss goals.

Anticipating that many patients may benefit from more frequent nudging and greater personalization of the behavioral support, the design team recommended 2 additional intervention components with these goals. Because more intensive services introduce additional implementation challenges and cost, we evaluated these components by offering them only to about half (n=42) of patients who had been randomized to receive, free of charge, CLS services. Specifically, for the first 12 weeks, each patient in the CLS arm was sent an automated weekly MyChart message with coaching tips customized to their use of the e-Scale in the previous week, whether they had made weight loss progress (defined as ≥0.5 pounds of weight loss from the first to last weight measure during the previous week), and whether they had enrolled in referral-based services offered by the fitness partner. A practice nurse used an EHR dashboard to monitor the entire CLS group and attempted to call patients to offer customized “step-up” telephone coaching if either (1) they had not used their scale in the previous week or (2) for 2 consecutive weeks they had not achieved ≥0.5 pounds of weight loss.

### Study Outcomes

The primary quantitative outcome was the percentage of patients achieving ≥5% weight loss from baseline at 6±3 months. Additional outcomes included the feasibility of automated trial processes (eligibility determination, enrollment, and randomization); the feasibility of pragmatic weight data capture from office visits and e-Scales; usage of intervention components; health care system costs to implement each intervention component; and patient perceptions regarding appropriateness, ease of access, ease of use, acceptability, desirability, credibility, and usefulness.

### Data Sources

EHR data spanning ±15 months of the launch of the trial provided information about patient baseline characteristics, service usage patterns, and metabolic and biometric outcomes, including body weight and height measured at office visits. BodyTrace e-Scales given to all study patients provided a secondary source of body weight data when weight data were missing in the EHR. The scale manufacturer reports accuracy within ±0.1 kg to a weight limit of about 150 kg (330 lbs) [30].

Multiple data sources were used to describe the usage of the different intervention components. Input from health care system leaders and study team members identified the specific personnel, supplies, contract services, and facility and administrative inputs needed to implement each intervention component. e-Scales provided data on the frequency of weight self-monitoring. Administrative data systems captured the number and type of contacts made between nurses and study participants. The partnering fitness network also provided data about patient participation in the 2 coaching support options and facility use.

Multiple data sources were also used to inform health care system costs to implement each intervention component, including personnel, supplies, technology, and other facility and administrative expenses. Staff members employed by both the health care system and the partnering fitness network were interviewed to estimate the amount of time they spent supporting different intervention activities over 6 and 12 months. Hourly salary costs of different personnel were collected from reports by the US Bureau of Labor Statistics. Costs of e-Scales, community intervention program fees, and development costs for health IT components were determined directly from invoice amounts. Maintenance costs for IT support were estimated from interviews with IT staff members, who were asked to estimate hours per week spent supporting the intervention components.

Patient-reported data were collected by semistructured telephone interviews with 15 randomly selected patients who were recruited beginning approximately 12 weeks after their enrollment in the feasibility trial (refer to the “Eligibility Criteria and Recruitment of Feasibility Trial Participants” subheading). Interviews assessed perceptions regarding the appropriateness, ease of access, ease of use, acceptability, desirability, credibility, and usefulness of the intervention components in helping the patient achieve health-related goals [11,31].

### Variable Construction for the Weight Loss Outcome

Analysis of body weight changes used data from both the EHR and e-Scales. The last EHR weight measure before each patient’s randomization date served as the baseline body weight, and the EHR weight measure closest to the date of randomization plus 6±3 months served as the follow-up weight. For patients who were missing such a follow-up EHR weight value, we estimated the 6-month value as one-half the difference between the baseline value and the EHR weight value closest to 12±3 months. For participants missing both a 6- and 12-month weight value in the EHR, we used the e-Scale weight value closest to 6±3 months. The percentage of weight loss at 6±3 months was calculated as: [baseline body mass (kg) – 6-month body mass (kg)] ÷ baseline body mass (kg). The dichotomous outcome of achieving ≥5% weight loss at 6±3 months was then determined for each patient. For patients missing all follow-up weight data, we assumed conservatively that they failed to reach 5% weight loss.

### Analysis of the Feasibility of Automated Enrollment and Randomization Procedures

Univariate and bivariate descriptive statistics were used to examine the time needed to accrue the targeted sample size and to assess representativeness by comparing the demographic and clinical characteristics of patients enrolled with those of the entire eligible patient population. We also compared the sample allocation and balance of demographic and clinical characteristics of patients in each treatment arm following the automated allocation procedure.

### Analysis of the Feasibility of Pragmatic Weight Data Capture

We evaluated the feasibility of weight outcome data capture from the pragmatic sources by examining proportions of patients with sufficient EHR or e-Scale data capture at the different time points of interest (baseline, 6±3 months, and 12±3 months).

### Analysis of Preliminary Data for Weight Loss Effects

We used chi-square tests to compare all patients randomized to CLS and BLS arms on the proportion that reached the dichotomous outcomes of any weight loss at 6 months and the intervention goal threshold of ≥5% weight loss at 6 months.

### Analysis of Patient Engagement in Intervention Components

We used descriptive statistics to summarize proportions of study patients who engaged in different intervention components (ie, e-Scale self-weighing, referral-based coaching resources, step-up telephone coaching by health care system nurses), as well as their volumes of use and timing of use relative to both study enrollment and the sudden start of the COVID-19 pandemic

### Analysis of Intervention Delivery Costs

We summarized the mean per-person intervention cost for each study arm, both overall and across categories of personnel, materials and supplies, and facility and administrative costs.

### Analysis of Patient Perceptions Toward Intervention Components

We conducted qualitative analysis on semistructured interview transcripts capturing patient perceptions regarding the appropriateness, ease of access, ease of use, acceptability, desirability, credibility, and usefulness of intervention practice components (Table S2 in Multimedia Appendix 1). Example themes and representative quotes were selected by group deliberation and consensus and are summarized in narrative format.

### Ethical Considerations

All study procedures were performed in accordance with the Declaration of Helsinki. Study procedures, including pragmatic enrollment and randomization under a waiver of written informed consent for research, were approved by the Northwestern University IRB (STU00207153). The pilot trial was registered at ClinicalTrials.gov (NCT03998046) and posted on June 25, 2019.

## Results

### Feasibility of Automated Trial Enrollment and Randomization Procedures

**Figure 1.**
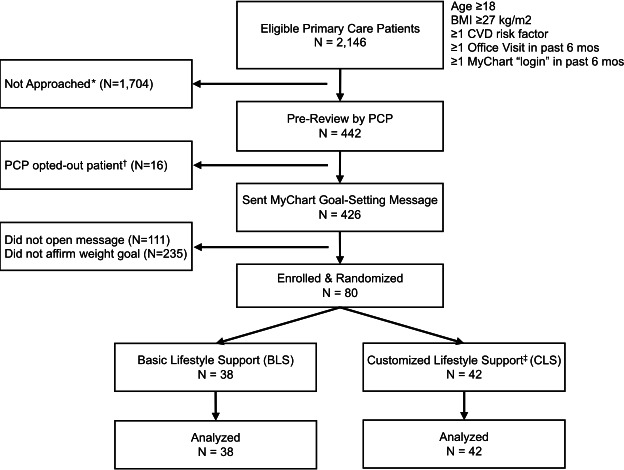
Feasibility trial flow. CVD: cardiovascular disease; PCP: primary care physician. * Batches of 100-200 patients were randomly selected from the eligibility list for pre-review by their physician before being sent a single MyChart message offering weight loss support; this procedure was repeated every 2 weeks until 80 patients had accepted the services; patients remaining on the list were not approached; † Prior to sending a MyChart message to eligible patients, PCPs asked to opt-out 16 (3.6%) patients from being offered services; ‡ Study patients who were randomized to receive “Customized Lifestyle Support” services received additional weekly email messages adapted to individual weight loss progress and step-up telephonic coaching by a nurse when they faced challenges (see text for details).

The eligibility list for the feasibility trial included 2146 patients with a mean age of 58.3 years and mean BMI of 33.5 kg/m^2^; 1295 (60%) were women, 783 (36%) were aged ≥65 years, 617 (29%) were non-Hispanic Black, 1089 (51%) were non-Hispanic White, and 184 (9%) were Hispanic. Over 6 weeks, the clinical data manager sent names of 442 randomly selected eligible patients to 34 primary care clinicians for prereview; clinicians asked that only 16 (4%) patients not be contacted. A single outreach MyChart message was sent to each of the remaining 426 patients, of whom 315 (74%) opened the message within 4 weeks, and 80 (19%) affirmed interest in the weight loss goal (Figure 1).

Demographic characteristics of the 80 participants (n=50, 63% women; n=24, 30% aged ≥65 years; n=23, 29% non-Hispanic Black; n=34, 43% non-Hispanic White; n=11, 14% Hispanic or Latino) approximated the characteristics of the overall target population, with slight over-representation of those identifying as Hispanic or Latino, non-Hispanic Black, or non-Hispanic Asian (Table 1). In the setting of relatively small sample sizes, trial patients randomized to CLS were more often than BLS patients to be older than 45 years, male, and Hispanic or Latino. CLS patients were also more likely to have prediabetes and a higher Charlson comorbidity score [32].

**Table 1.** Baseline characteristics of study patients.

Baseline characteristic^a^	Eligible, approached (n=426)	Affirmed weight goal, enrolled (n=80)		Basic lifestyle support (BLS; n=38)	Customized lifestyle support (CLS; n=42)	Approached and completed interview^b^ (n=15)
Age group (years), n (%)						
18‐44	61 (14)	12 (15)		8 (21)	4 (10)	0 (0)
45‐64	230 (54)	44 (55)		18 (47)	26 (62)	12 (80)
Age 65+	135 (32)	24 (30)		12 (32)	12 (29)	3 (20)
Sex, n (%)						
Female	234 (55)	50 (63)		26 (68)	24 (57)	10 (67)
Male	192 (45)	30 (38)		12 (32)	18 (43)	5 (33)
Race or ethnicity, n (%)						
Hispanic or Latino	44 (10)	11 (14)		3 (8)	8 (19)	4 (27)
Non-Hispanic Black	113 (27)	23 (29)		12 (32)	11 (26)	6 (40)
Non-Hispanic Asian	14 (3)	6 (8)		2 (5)	4 (10)	0 (0)
Non-Hispanic White	217 (51)	34 (43)		16 (42)	18 (43)	4 (27)
Other	38 (9)	6 (7)		5 (13)	1 (2)	1 (7)
Qualifying diagnoses, n (%)						
Prediabetes	128 (30)	30 (38)		19 (50)	11 (26)	6 (40)
Diabetes	138 (32)	19 (24)		6 (16)	13 (31)	5 (33)
High blood pressure	324 (76)	62 (78)		29 (76)	33 (79)	13 (87)
Dyslipidemia	341 (80)	61 (76)		27 (71)	34 (81)	11 (73)
Charlson score^c^, mean (SD)	1.34 (1.79)	1.33 (1.97)		1.13 (1.70)	1.50 (2.19)	1.13 (1.46)
Baseline risk factor levels, mean (SD)^d^						
System International (SI) Units						
Mean weight, kg	95.0 (15.8)	94.3 (17.2)		95.1 (18.4)	93.6 (16.2)	95.6 (18.5)
Mean BMI, kg/m^2^	33.2 (5.6)	33.6 (6.1)		34.1 (7.1)	33.1 (4.9)	34.7 (8.5)
Hemoglobin A1c, mmol/mol	44.5 (11.5)	43.8 (12.6)		42.4 (9.8)	45.2 (14.8)	42.5 (6.7)
Systolic blood pressure, mm Hg	131 (15)	135 (18)		137 (18)	133 (18)	135 (20)
Total cholesterol, mmol/L	4.69 (1.15)	4.79 (1.17)		4.74 (1.06)	4.84 (1.27)	5.20 (1.18)
HDL^e^ cholesterol, mmol/L	1.33 (0.36)	1.42 (0.35)		1.45 (0.40)	1.39 (0.31)	1.38 (0.30)
Non-HDL cholesterol, mmol/L	3.36 (1.07)	3.37 (1.06)		3.29 (0.91)	3.45 (1.18)	3.82 (1.19)
Conventional units						
Mean weight, lbs	209.0 (34.8)	207.5 (37.8)		209.2 (40.5)	205.9 (35.6)	210.4 (40.8)
Hemoglobin A1c, %	6.2 (1.1)	6.2 (1.2)		6.0 (0.9)	6.3 (1.4)	6.0 (0.6)
Total cholesterol, mg/dL	181.3 (44.3)	185.4 (45.3)		183.2 (41.0)	187.3 (49.2)	201.1 (45.4)
HDL cholesterol, mg/dL	51.3 (14.1)	54.9 (13.7)		56.1 (15.5)	53.8 (12.0)	53.4 (11.7)
Non-HDL cholesterol, mg/dL	130.0 (41.4)	130.4 (40.9)		127.1 (35.2)	133.5 (45.6)	147.8 (45.9)

a Sex and race or ethnicity are based on self-reported values stored in each patient’s electronic chart. Weight, blood pressure, hemoglobin A1c, and cholesterol values are based on the last recorded value within the past 12 months in each patient’s electronic chart. BMI is based on the last recorded weight and height in each patient’s electronic chart. Some column percentages may not sum to 100% due to rounding.

b Thirty of the 80 trial participants were randomly selected and approached for interviews to recruit 15 respondents.

c Charlson comorbidity score is a weighted score based on the number of comorbid diagnoses.

d Values are based on electronic health records. One hundred percent of patients had a weight and systolic blood pressure value in the past 12 months. A1c values were available for 338, 67, 33, 34, and 13 patients, and cholesterol values were available for 418, 78, 37, 41, and 14 patients in the eligible, enrolled, BLS, CLS, and interviewed groups, respectively.

e HDL: high-density lipoprotein.

### Feasibility of Pragmatic Weight Data Capture

Despite lower-than-usual office visit rates during the early phase of the COVID-19 pandemic, 44 (55%) of feasibility trial patients had an office weight measure recorded in the EHR at 6±3 months from their trial enrollment. Among the remaining 36 patients, 26 (72%) had a weight recorded at 12±3 months, and of the remaining 10 patients, 7 (70%) had an e-Scale weight received at 6±3 months. Thus, our pragmatic data capture strategy enabled estimation of a 6-month weight change for 77 (96%) of all trial participants. The average time between trial enrollment and the weight used for the 6-month data analysis was 184.5 days.

### Analysis of Preliminary Weight Loss Effects

Overall, 48 (60%) of 80 participants had lower weight at follow-up than at baseline; 12 (15%) exhibited a weight loss of ≥5% of baseline weight. The sample size for this feasibility study was not designed to test differences in weight loss goal achievement between randomized treatment arms, and there was no statistically significant difference between CLS and BLS (*P*=.85).

Using e-Scale data and assuming that 11 of the 80 patients who did not self-weigh also did not lose weight, 28 (35%) of all participants achieved ≥10 lbs of weight loss (ie, the initial goal recommended by the MyChart message), and 24 (30%) achieved ≥5% of weight loss at some point during the 12-week automated messaging phase. Thus, half of the patients who achieved a 5% weight loss during the 12 weeks of active support maintained the loss at 6 months.

### Patient Engagement in Intervention Components

All except 3 (4%) patients were able to initialize their e-Scale and register a baseline weight. Over the 12-week MyChart messaging phase of the intervention, 11 (14%) study patients did not use their scales for self-weighing, 7 (9%) participated in weighing in some but not all weeks, and 62 (78%) engaged in self-weighing during all 12 weeks. Self-weighing daily (5 to 7 days per week) was performed by 26 (33%) patients during all 12 weeks; more patients assigned to CLS (customized weekly messaging) performed daily self-weighing in all weeks (18/42, 43% patients) compared with those in BLS (8/38, 21%; *P*=.06).

Overall, 36 (45%) patients enrolled in one of the two referral-based coaching platforms offered by the fitness industry partner, 14 (37%) in the BLS arm and 22 (52%) in the CLS arm (*P*=.18). Among all who enrolled, 22 (28%) elected the face-to-face option and 14 (18%) elected the fully remote delivery option. Patients enrolled in these services between 15 and 77 days after receiving the initial outreach MyChart message. Access to fitness facilities (ie, the face-to-face delivery option) was disrupted by a COVID-19 pandemic stay-at-home order beginning the third week of March 2020. At that time, patients had been enrolled in the referral-based services for 19 to 75 days; the 22 patients in face-to-face support had completed an average of 3 (range 1-6) individual coaching sessions and attended a partnering fitness facility 0 to 24 times. The 14 patients receiving fully remote support had completed an average of 4 (range 2-5) individual coaching sessions before the pandemic and an average of 3 additional (range 0-5) sessions afterward. Among patients randomized to CLS, 41 (98%) received at least one step-up telephonic nurse coaching call; 19 (45%) received ≥5 nurse calls (maximum 11 calls received) over the 12-week intensive support phase.

### Intervention Delivery Costs

Direct medical costs associated with offering each intervention component are summarized in Table S3 in Multimedia Appendix 1. Mean health care system costs to offer the intervention as delivered during the feasibility trial were estimated at US $335 per person over the first 6 months (US $284 for BLS patients; US $382 for CLS patients) and US $150 per person over the second 6 months (US $124 for BLS; US $174 for CLS patients).

### Patient Perceptions Toward Intervention Components

Among the 80 feasibility trial participants, 30 were randomly selected and approached, of whom 15 agreed to complete a telephone interview; 11 were unreachable, 5 declined participation, and 2 asked to be recontacted at a future time. Respondents included 5 men and 10 women, with demographic and clinical characteristics generally representative of the overall target population (Table 1). Patient perceptions are summarized below; representative quotes and considerations for design refinements are organized in Table S2 in Multimedia Appendix 1.

Most patients described the MyChart messages and website resources as generally easy to understand and navigate. However, some reported feeling frustrated or less engaged by not having a clear understanding upfront for how the different intervention components were coordinated or should be used. Trust in the person or organization providing lifestyle support services emerged as an important theme. Some patients expressed being distrustful, in general, when offered resources for “free” from another organization, recommending that it be made very clear that their doctor or the health care system was recommending and coordinating the activities. Others indicated that they had not initially realized they could access dietary and physical activity coaching at the partnering fitness organization and were unsure how to engage with the health care system nurse if they had problems.

Nearly all patients reported that an initial weight loss goal of about 10 pounds in 10 weeks was appropriate and achievable. Some expressed interest in larger weight loss goals and access to services beyond 10 to 12 weeks. Many patients reported that their weight loss goal was motivated by other health or social goals, such as reducing the need for more pills to treat high blood pressure.

Several patients highlighted the importance of supportive accountability in successfully changing behavior. They emphasized the practice nurse, registered dietitian, and physical trainers as key sources of supportive accountability, more so than the automated messages. Patients expressed strengths and limitations of the automated messages, highlighting that the messages were a helpful reminder or source of encouragement when making progress, but were insufficient or even demoralizing when not making progress.

Many patients viewed self-weighing as an additional source of accountability. Some expressed interest in more elaborate e-Scales or integration with other technologies, such as smartphone apps that provide immediate and customized feedback. Other patients indicated that they preferred support from a member of the health care or fitness center team. Generally, patients expressed frustration when they viewed e-Scales (and self-weighing) as a direct source of support**,** rather than as a means for health care providers and coaches to track and customize their support. Importantly, some viewed daily weighing as a source of stress and felt that, no matter how closely they followed diet and physical activity plans, their daily weight remained unpredictable. Several patients highlighted low expectations for success stemming from negative experiences with previous weight loss attempts. Those patients recommended a greater focus on recognizing and addressing such expectations, as well as de-emphasizing a focus on the daily weight readings as a sign of success or failure, while offering more immediate support beyond automated messaging. Relatedly, some patients highlighted that emotions have a larger influence on dietary behaviors than reminders to eat healthy. Those patients recommended a greater focus on acknowledging and addressing emotions both as drivers and consequences of attempts to lose weight and change diet.

Finally, all patients indicated that the abrupt start of the COVID-19 pandemic presented a profound barrier to healthy behaviors and weight loss. Many indicated frustration with becoming more sedentary, adopting “stress eating” behaviors, and gaining weight. The pandemic restrictions eliminated access to fitness facilities, which further contributed to frustration.

## Discussion

### Principal Findings

This preliminary study was conducted to demonstrate the feasibility of several pragmatic practice components and implementation strategies for primary care offices to support the core components of ILIs. We also sought to demonstrate the feasibility of capturing and analyzing weight data from 2 secondary sources, the EHR and e-Scales, as an important biometric outcome for future research. After a single outbound MyChart message was sent to encourage a weight loss goal by 426 overweight or obese adults with cardiovascular risk factors, 315 (74%) opened the message, and 80 (25%) of those who opened it affirmed interest in receiving support services to lose weight. Among those 80 patients, 24 (30%) achieved ≥5% weight loss in the first 12 weeks and, despite the disruptive impacts of a pandemic, 12 (15%) still exhibited ≥5% weight loss at 6 months, at a time in history when weight gain was common [33-35].

Weekly “nudging” of patients with a customized MyChart message plus step-up telephone coaching by a practice nurse increased participation in daily self-weighing (43% vs 21% of patients through 12 weeks) and enrollment in referral-based lifestyle support resources offered by a partnering fitness organization (52% vs 37%). Although this small feasibility trial did not demonstrate whether patients are more likely to reach weight loss goals when provided with these more customized services, this approach warrants further study.

### Relationship to Other Past Research

Our work is consistent with other recent studies of pragmatic, technology-facilitated lifestyle interventions in primary care settings [13-17], but we are unaware of other examples where the intervention components have been fully embedded within an EHR system. Our approach used design thinking with service professionals, health care system leaders, health payers, and researchers to create practice components that not only are evidence-based but also desirable to implementers, technically feasible, and financially sustainable [11]. Design prototypes were iteratively pretested and refined with stakeholder collaboration, and a small feasibility trial demonstrated strong promise for both effectiveness and sustainability.

Our work also builds on past research evaluating referral linkages between health care systems and community-delivered ILIs [36-39]. However, most past research focused on referrals to a comprehensive program bundle, such as the National Diabetes Prevention Program, often offered in a single format or location [18,40]. Offering a multicomponent program such as the National Diabetes Prevention Program requires considerable community organizational capacity and financial investment, which imposes implementation barriers [41,42], particularly when enrollment is low or third-party payment is limited. A unique aspect of our approach was to distill ILI programs down to core components and to develop implementation strategies for each component to minimize demands placed on the implementing organization [22]. The resulting approaches were designed to help the partnering health care systems achieve existing goals relating to US Preventive Services Task Force recommendations for linking overweight or obese adults with cardiovascular risk factors with evidence-based behavioral interventions, while minimizing interference with existing, busy clinical workflows or placing more work on clinicians. We also considered how to offer the resulting services using an array of different channels (eg, MyChart, telephone, smartphone application, and fitness facility) to maximize reach by appealing to different segments of the target population. By leveraging existing clinical personnel with skills and incentives to support behavioral support services, linkages with extant service organizations that offer ILI resources, and EHR technologies that are now available in most health care settings, these pragmatic approaches, if effective, could have high potential for reproducibility in other settings.

### Implications for Practice and Further Research

Conceptually, the development of discrete implementation strategies for individual ILI components has appeal if they successfully minimize workflow disruptions, distribute costs of the new activities across different partners, and map costs to existing pathways for health payer reimbursement. Possible disadvantages are that the individual components may not prove as effective in isolation, and additional strategies must be developed to coordinate staff roles, technologies, and patient transitions among the practice components. Some patients may still face access barriers if, for example, referral-based resources are limited or health payers do not provide reimbursement for remote weight monitoring or lifestyle counseling activities by nonphysicians [43,44]. Although our approach offers lower-technology intervention options, such as nurse-delivered telephone counseling and facility-based resources offered by a partnering fitness organization, these interventions may still have different effects based on technology preferences and literacy. It will be important for future research to evaluate these dimensions of implementation in the context of a larger and more definitive effectiveness trial.

### Conclusion

This preliminary research indicates that strategies to implement ILI core components are feasible for primary care practices and generally acceptable to patients. Further research should seek to optimize implementation strategies to maximize feasibility, acceptability, sustainability, and reach, as well as to demonstrate effectiveness and cost-effectiveness compared with alternative service models. Engaging stakeholders in cocreation of these strategies holds promise to improve both implementation and reach of ILI components in new ways that achieve population health and health equity for millions of Americans who are currently overweight or obese and already engaged by the health care sector.

## Supplementary material

10.2196/58722 Multimedia Appendix 1 Intervention design details.

10.2196/58722 Checklist 1 CONSORT-eHEALTH checklist (V 1.6.1).

## Abbreviations

BLS: basic lifestyle support
CLS: customized lifestyle support
CONSORT-EHEALTH: CONsolidated Standards Of Reporting Trials for Electronic Health
e-Scale: electronic scale
EHR: electronic health record
ILI: intensive lifestyle intervention
IRB: Institutional Review Board
