# Supplementary material for: Hybrid Health IT and Telehealth–Delivered Behavioral Weight Loss Services for Primary Care Patients With Cardiovascular Risk Factors: Intervention Component Design and Pragmatic Randomized Feasibility Trial
Source: JMIR Mhealth Uhealth. 2025 Oct 22;13:e58722. doi: 10.2196/58722 (PMC12543212; doi:10.2196/58722)
Supplement: Multimedia Appendix 1 [file mhealth-v13-e58722-s001.docx]

**Contents**

The appendix provides additional details regarding the intervention design and is organized into 3 sections:

| **Section 1:** **Stakeholder-informed Design Themes**  Table S1: Design Considerations and Strategies Emerging from Stakeholder Meetings and Interviews  **Section 2:** **Description of Resulting Intervention Goals and Practice Components**  **Section 3:** **Additional Information Regarding Acceptability and Cost of Practice Components**  Figure S1. Timeline for Intervention Design, Delivery, and Evaluation  Table S2: Patient Perceptions Regarding Intervention Components and their Design Implications  Table S3: Estimated Delivery Costs from the Health System Perspective  **Section 4: Patient Semi-Structured Interview Guide** |
| --- |

**Section 1: Stakeholder-Informed Design Themes**

Between September 2016 and May 2018, we engaged numerous stakeholders individually and in groups to consider published research evidence for intensive lifestyle intervention (ILI) core components[1-3] and to co-create implementation strategies for the feasible and sustainable delivery of each core component in busy primary care practice settings. Research staff members organized stakeholder meetings, kept detailed written notes, verified recommendations and action steps with participants, and integrated ideas and recommendations into an evolving array of design themes and concepts that were shared with the primary implementers and other stakeholders and were used to guide intervention prototypes, pretests, and refinements.[4] Design themes emerging from this process are described below.

Using the Electronic Health Record System to Integrate and Coordinate ILI Components

Clinicians reported that almost all professional communications, most patient communications, and most tools for coordination and monitoring of other health care services are now supported directly by the electronic health record (EHR). Thus, unless new ILI practice components align within existing EHR tools and workflows, they are unlikely to be used routinely and efficiently. They recommended implementation strategies that *take advantage of EHR data, patient communications, clinician communications, decision support tools, and panel or population management tools* that help professionals to target patients in different ways, based on their individual engagement in services and progress towards goals. However, several clinicians admitted having a limited understanding of what might be possible to offer patients directly within MyChart, and how many would engage in ILI components when offered to them in a MyChart message. They cautioned that older and lower income patients, as well as those living in low resource community areas might be less likely to use technologies such as MyChart or engage in services offered by a local fitness organization, even if provided free of charge. Stakeholders recommended *monitoring engagement* for each patient and *being proactive to call patients who do not engage or make progress towards goals* and to offer those patients alternative forms of support with other channels, such as telephone, video, or brief office visits. They also recommended *assessing the usability and acceptability of technologies, both for clinical staff and for patients*.

Framing Weight Loss Goal-setting as a Means to Improve Cardiovascular Risk Factors

Most clinicians involved in the design process had views regarding the appropriateness of different approaches for advising and assisting patients to set a weight loss goal. Many thought that some patients may view the process as off-putting or stigmatizing, unless the weight loss goal was presented as means to feel healthier or to avoid or minimize the need for “medical” interventions such as medications needed to lower blood pressure or blood glucose. Clinicians also voiced strong beliefs that encouraging patients to adopt a weight loss goal would need to be coupled with access to support resources that could help them achieve and maintain diet and activity changes. Most clinicians were not confident in their own abilities to provide effective support for these behaviors but believed it would be appropriate for health systems to offer such services using technology, dedicated non-physician personnel or teams, or by referral to other partnering organizations. Most believed that weight loss goal setting, particularly if it were encouraged by an unprompted MyChart message, would be more acceptable to patients if it were *framed as a means to improve cardiovascular risk factor control*, including as a means to minimize the need for medications to control those risk factors. Stakeholders also believed that targeting patients with mildly overweight BMI’s increased the possibility that weight loss might not be appropriate or acceptable to some patients; they recommended targeting those with BMI ≥27 kg/m2.

Balancing Technologies with Coaching Roles by Non-Physicians

Stakeholders viewed time limitations of clinicians and staff members as the biggest challenge for implementing new ILI practice components. Most healthcare encounters involve a physical office visit between one patient and a physician, and health insurers provide reimbursement to the health system based on the complexity or time spent during that visit. In general, clinicians and health system leaders thought it would not be feasible for physician office visits to focus solely on healthy weight loss or for each patient to return every 1 to 4 weeks for longitudinal coaching visits. They recommended *against strategies that would require additional activities to take place during existing office visits, particularly involving physicians*. Stakeholders expressed interest in *using technology to minimize the need for professional support*, particularly for some patients who might achieve lifestyle goals with simple reminders and automated forms of support alone. They also believed that some *coaching support could be provided effectively by non-physicians, such as nurse practitioners, care management nurses, or trained professionals outside the practice*.

Assigning Nurses Efficiently to Perform “Step-up” Coaching Roles

Clinicians recommended close monitoring of individual patient progress and *“stepping up” levels of support using more “hands on” and individualized approaches*, in which clinical personnel reach out directly to patients who do not engage or make progress after receiving “lower touch” strategies. *Nurses were viewed as being the most appropriate existing primary care team member* to support patients who need more help with goal setting, problem solving, or choosing from different coaching options. However, many stakeholders expressed concerns that nurses may still need to perform these roles by telephone, video, or in email communications occurring outside office visits. Only select members of the nursing staff currently performed similar roles for other patients, usually in the context of providing care or case management services for patients with complex chronic conditions or recent high utilization of hospital or ED services. Health system leaders and nurse managers recommended *evaluating strengths and weaknesses of assigning “step up” coaching roles to nurses, including the amount of time and personnel cost associated with such a role*.

Expanding the Role of Referral-based Coaching Services that are Patient-centered

In a very general sense, many clinicians thought it appropriate to provide patients with referrals to dietary and physical activity resources that already exist outside of the health system. However, health system leaders also questioned the appropriateness of offering services by referring patients away from the health system. They were willing to explore *inter-professional partnerships* with outside organizations *only if those partners were clearly better suited to deliver specific ILI components*, and if the arrangement was *acceptable to patients*. Clinicians viewed *registered dietitians* as an ideal source of nutritional coaching, but they pointed out that dietitians are not employed currently in most primary care settings. They liked the idea of *collaborating with an established fitness organization in the region that employed both registered dietitians and other health educators*. However, formal agreements with non-health care organizations to deliver preventive services remain unusual, and stakeholders raised questions about how to ensure quality and affordability of referral services. Clinicians advised strategies that *minimize costs to patients*. They also advised keeping choices simple; *if patients are offered multiple service options, they should be offered support to help them choose,* possibly by a clinical staff member, by using MyChart, or with decision tools found on a simple website managed by the health system. Clinicians recommended monitoring the quality and outcomes of practice components delivered by partner organizations to understand if they are *acceptable to patients and effective*.

Using the Evaluation to Confirm Acceptability and Sustainability of each Component

Clinicians and staff members recommended that health system leaders or the research team use existing practice meetings and also offer brief training sessions with key service professionals to ensure they understand any new responsibilities, use of new technology applications, and how their performance will be measured. They also raised concerns that patients will approach staff with questions about the content of MyChart messages, use of electronic scales (eScales), and services offered by partner organizations; they recommended *preparing staff with simple answers for any questions that might be voiced by patients*. Health system leaders recommended that the new ILI practice components be *positioned as strategies to achieve other existing quality improvement or pay-for-performance goals*, such as improving diabetes care and outcomes or other CVD risk factor control. Leaders also recommended *evaluating the time needed for different personnel* to support the ILI components, *whether team members believed they were prepared* adequately to perform the role and could perform it routinely, and *whether health insurers were likely to reimburse* the health system or fitness partner organization for services delivered.

Stakeholders’ views were incorporated as design elements into ILI practice components and implementation strategies. Key themes of recommendations made by stakeholders are summarized in **Table S1**.

**Table S1.** Design considerations and strategies emerging from stakeholder meetings and interviews.

|  |  | Considerations for sustained implementation | | |
| --- | --- | --- | --- | --- |
|  |  | **Acceptability** | **Appropriateness** | **Feasibility** |
| ILI Dimensions and Core Components | General | - Health systems should deliver components directly unless not feasible or acceptable for staff - Don’t refer patients to outside services that require fees | - Engagement using health IT might not be appropriate for all patients - For patients who do not engage or make progress with IT approaches, explore telephone and video encounters with non-physician staff | - Avoid delivery of new practice components during primary care office visits - Simplify new tasks that practice team members must perform - Coordinate interprofessional communication, initial patient communication, and progress monitoring within the EHR |
|  | Goal Setting | - Ask patients how they view being offered weight management services from a MyChart message | - Automated interventions should target adults with BMI≥27 & ≥1 CVD risk factor - Frame weight loss as means to improve control of CVD risk factors - Encourage weight loss at a rate that is achievable and clinically meaningful for most patients (0.5 to 2.0 pounds per week); provide resources to support success | - Target patients between rather than during office visits - Begin with MyChart; monitor and offer “lower tech” alternatives for those who do not engage via IT - Frame an “initial” goal that most patients perceive as feasible; from prior research, an initial goal of 10 lbs over 10-12 weeks (~1 pound per week) will be technically feasible to support - Patients with larger weight loss goals may be encouraged to “begin with the first 10 lbs,” and to build on success to reach their larger goal beyond 12 weeks |
|  | Self-Monitoring Support | - Ask patients about the usability of the eScale - Ask how they feel about a scale transmitting their weight measures to their electronic chart | - Ask patients if self-weighing helps them feel supported and accountable - Ask patients if self-weighing causes distress or frustration | - Leverage increasing use (and payment) for remote patient monitoring - Provide a wireless scale that transmits data into the patient’s electronic chart |
|  | Longitudinal Coaching | - Ask patients about the usability of MyChart - Ask if they view automated messaging as helpful & supportive | - Ask patients if MyChart messages are easy to access - Ask patients if automated messages cause distress or frustration | - Begin with MyChart; monitor and offer ‘human’ alternatives for those who do not engage or make progress - Offer free-of-charge access to services provided by the fitness partner (both face to face and ‘virtual’ coaching options would be ideal) |
|  | Coordination | - Ask patients how they feel about the health system working with a fitness organization to provide services | - Monitor handoffs between health system and fitness partner to ensure services are reliable and consistent - Use existing channels to engage, prepare, & support staff to perform new roles | - Use existing EHR messaging platforms (ideally expanded to enable access by coaches at the fitness center) - Use EHR “population” health reporting tools and dashboards to target patients with different services based on progress - Document remote patient monitoring (RPM) and telemedicine education for RPM results in ways that align with health payer reimbursement |

**Section 2: Description of Resulting Intervention Goals and Practice Components**

By integrating the themes that emerged from stakeholder meetings and interviews, the intervention design team framed the following strategic goals for implementation of ILI core components: (1) targeting adults based on BMI ≥27 kg/m2 PLUS ≥1 cardiovascular risk condition (prediabetes, type 2 diabetes, hypertension, or abnormal blood cholesterol); (2) supporting patients to commit to an initial weight loss goal of 10 pounds (about 5 kg) over 10-12 weeks; (3) providing support and feedback for self-monitoring and self-regulation of diet, physical activity, and weight change; and (4) linking patients with forms of longitudinal coaching support that can be refined based on social and structural conditions, personal preferences, self-efficacy, and progress towards achieving goals. These four goals were considered as design parameters during the development and pretesting of implementation strategies.

Members of the health system’s technology support team determined technical feasibility and developed prototypes for testing and refinement. Technology components included the following: (A) new EHR reports and dashboards to enable clinical personnel to identify and monitor high risk patients who may benefit from primary care weight management services; (B) a brief, theoretically-grounded weight loss goal-setting message to be sent to multiple patients simultaneously using the EHR's secure patient messaging tools; (C) an electronic scale (eScale) that enables patients to self-monitor their weight and clinical personnel to remotely monitor each individual as weight data are transmitted back to the electronic chart; (D) automated EHR algorithms that analyze the remote weight monitoring data to categorize patients into groups based on their engagement in services and progress towards a weight goal; and (E) new applications of EHR technologies to encourage and provide each patient with customized lifestyle coaching. Three complementary IT strategies were developed to encourage access to different forms of lifestyle coaching. First, EHR algorithms were developed to send each patient a weekly motivational message, which was generated and sent to each patient automatically via MyChart; each message was customized using remote weight monitoring data to offer different forms of encouragement based on engagement in daily weighing and weekly progress towards the weight loss goal. Second, each MyChart message incorporated a “behavioral nudge” to remind and encourage each patient to engage free-of-charge in longitudinal, referral-based dietary and physical activity behavioral support resources offered by a partnering fitness organization in either face-to-face or “virtual” formats. Finally, EHR “panel management” tools were adapted to prepare and remind practice nurses to offer step-up telephonic coaching to any patient who was not engaged in self-weighing, was not making steady weight loss progress, or who requested more assistance. More detailed descriptions of each component are found below.

Reporting Tools and Dashboards to Support Clinical Personnel Roles

A refreshable list of patients eligible for services was generated using the EHR's “Reporting Workbench” tool, which allows clinicians and staff to define specific patient parameters (e.g., age ≥18; BMI ≥27; ≥1 CVD risk factor) from data available in the EHR and generates an electronic report of all patients meeting those parameters. The report tool also enables providers to select one or more patients on the list to receive a message delivered in MyChart. Design of the initial “goal setting” message is described below.

Using similar EHR reporting functions, the health IT team also designed a dashboard to display progress metrics for all patients assigned to receive intervention support. The dashboard enabled clinical personnel to categorize patients based on 3 monitoring parameters: (1) engagement in self-weighing for the past week; (2) reaching a minimum weight loss threshold in at least one of the prior two weeks; and (3) enrollment in one of the referral-based coaching platforms offered by the fitness organization partner. An existing care management nurse employed by the health system was assigned to review the dashboard weekly and to initiate outreach contact by phone or by MyChart message to offer “step up” coaching in an alternative telemedicine format. Members of the nurse team already used EHR tools for “panel management” of other patient populations and had been trained in motivational interviewing to offer individualized coaching in lifestyle and disease-related behaviors.

The EHR panel management tools developed to support nurses in coordinating targeted weight management services also summarized each patient’s goal weight, progress to date, notes regarding each patient’s lifestyle action plan, and indicators for whether they had engaged in longitudinal coaching offered by the fitness partner. Each week, the dashboard specifically identified patients who were not self-weighing or not making weight loss progress for the preceding 2 weeks.

Goal-Setting Message

The initial MyChart patient message communicated the following: (1) modest weight loss and regular physical activity can improve blood pressure, cholesterol, and blood glucose;[1, 2] and (2) adults are 4 to 6-fold more likely to reach a weight loss goal if they (a) set a weight goal that is meaningful but achievable; (b) weigh themselves daily to track progress;[5] (c) receive expert coaching advice based on progress.[1, 6] The design team did not wish to discourage patients from setting larger weight loss goals, but pragmatic concerns were raised about overburdening primary care staff to provide highly customized, “human-mediated” goal-setting support for a very large population at risk. There was also a concern regarding technology constraints for programming of highly individualized goal-setting algorithms into the EHR system. Balancing these concerns, the resulting strategy was to encourage an initial goal for the first 10-12 weeks, with weight loss at an evidence-based rate of 0.5 to 2 lbs per week. The team created a message that encouraged an “initial” goal of about 10 pounds in 10 weeks. Patients who were encouraged by the message but wished to discuss the goal more were able to reply to the message or request a telephone call with a nurse educator in the practice.

Patients who were interested in setting the initial weight goal were instructed to click a link in the patient message, which prompted a brief “MyChart Survey” that collected patient responses to two brief questions: “Are you ready to commit to an initial weight loss goal of 10 pounds?” and “Will you be available in the Chicagoland area to work towards this goal over the next 10 weeks?” An EHR algorithm categorized all patients who answered “yes” to both questions as “enrolled,” which triggered a series of routines to initiate the additional practice components described further below.

Remote Weight Monitoring

Upon enrollment of a patient in weight management support services, the EHR system prompted a practice nurse to ship each patient a BodyTrace electronic scale (eScale).[7] Scales were pre-purchased by the health system, and came tagged with a unique device ID; the manufacturer kept a record of all device IDs assigned to the health system. When processing the “scale order,” the nurse was prompted to enter the unique ID number, found on the back of the scale. The EHR then associated the scale ID confidentially with the patient to which it was assigned. Once turned on, the eScale used existing cellular networks to transmit the time and value of each weight measurement, along with the unique scale ID to a secure server operated by the manufacturer. Nightly, a file with all deidentified data received from eScales with IDs “assigned” to our health system was transferred to the health system data warehouse, and an automated routine used the scale ID to assign each weight value to a unique patient and to store that data within their electronic chart.

Scales were shipped to patients with simple pictographic instructions that guide the user to register 3 initial weight readings, which were then used by a separate EHR “routine” to compute a baseline weight. Patients were instructed to perform all home weight readings consistently, typically before the first morning meal and after voiding. The health system IT team created an automated “data cleaning” routine, which performed range checks on each weight value to determine if it was likely a “valid” reading from the same person. Each week, the EHR routine saved all “valid” weight data and classified each person first by whether they had performed self-weighing on 0 days, 1 to 4 days, or 5 to 7 days (i.e., “daily”). The last valid weight in each week was used to calculate the weekly weight change, and each patient was classified as “making progress” (i.e., at least 0.5 pounds of weight loss in the past week) or not making progress. These data were stored in the patient’s chart and used to update the “panel management” dashboard (above), as well as to trigger additional automated Mychart messages (below). To keep primary care clinicians aware of progress, the daily weight values for each patient were also stored as flowsheets, which were transmitted to the clinician’s EHR “inbox,” following existing workflows for routing lab results and communications among clinicians.

Customized, Weekly Coaching Messages Delivered Automatically via MyChart

Automated coaching messages were designed at a 4th-grade reading level and aimed to provide a source of supportive accountability while encouraging two goal behaviors under one’s immediate control: (1) daily self-weighing and (2) engagement in additional forms of expert coaching services to support healthful diet and activity changes.

Wellness program specialists at the partnering fitness organization collaborated to develop longitudinal lifestyle coaching resources that embody the core components of evidence-based ILI using two different delivery formats. The first format was face-to-face, including individualized nutritional and physical activity goal setting and coaching, plus full fitness facility access at 11 facility locations. The second format included the same nutritional and physical activity coaching with “virtual” access via a video smartphone or computer application or by telephone calls. The computer/smartphone application also provided access to behavioral tracking tools and a suite of instructional videos for healthy food selection and preparation, as well as moderate intensity physical activities that could be performed by individuals within their own homes or in an outdoor setting with little to no equipment. Participants engaging in either format were encouraged to participate in one-on-one nutritional or physical activity coaching sessions at enrollment and during weeks 2, 4, 6, 8, 12, and then every 8 weeks through the end of 1 year.

To support choosing and enrolling in one of these two coaching resources, automated MyChart messages provided each patient with a “coupon code” and clickable link to a web page that provided simple summaries of the components and differences between each program with a single “enroll me” button that helped enroll the patient free of charge by entering their coupon code. Patients were offered a phone number to call at the health system if they had questions or needed more help deciding. For those not interested in these two coaching options, the website also included links to other low- or no-cost physical activity or weight management resources in the region. For patients who selected one of the two coaching service options, the fitness organization reached out within 2 business days to assist them to get started. The fitness organization used the coupon code to validate patients’ eligibility and to “bill” the health system for their intervention costs. The health system supported these costs for 12 months from the date of each patient’s enrollment. Since the health system generated the unique coupon code for each patient, it also served as a patient identifier that enabled the primary care practice team to monitor engagement in these coaching resources for each individual.

Pre-testing and Refinement of Intervention Components Prior to the Pilot Trial

Prior to pilot testing, technical aspects of IT workflows (i.e., back-end coding; deployment of prompts, alerts, or messages into the front-end of Epic applications) were developed, deployed in a developmental test setting (i.e., offline) and then pre-tested in controlled Epic “test patients” and other settings before being put into full deployment. Limited manual pretests were performed with 2-3 clinicians to ensure comprehension and usability of Epic message prompts and staff inbox messages that were designed to trigger specific new actions, such as a nurse entering a new order for a remote scale to be linked to a patient’s medical record and shipped. Both provider and patient message drafts were written by members of the study team with expertise in patient communications, behavioral message design, and clinical communications. Because different messages were drafted to align with the different categories of weekly response data (e.g., did versus did not weigh in the past week; weighed less than daily; weighed daily; last weekly weight was at or above weight goal versus last weekly weight was not at or above weight goal), the technical aspects of the messaging logic were reviewed by 2 analysts to ensure response data were received, processed and “cleaned” automatically by the back-end data warehouse routines, and resulted in the correct message being deployed. The draft messages were then reviewed by multiple other staff and clinicians and revised to improve readability and comprehension. After the fidelity of the technical workflows, message readability, and technology usability had been pretested, all modules were deployed into the “live” Epic interfaces prior to initiating the pilot trial.

**Section 3: Additional Information Regarding Acceptability and Cost of Practice Components**

Following iterative phases of intervention design, prototyping, and refinement, the resulting ILI practice components and implementation strategies underwent further feasibility testing in the small pilot trial involving 80 patients. The general timeline for intervention design, delivery, and evaluation are depicted in **Figure S1**.

**Figure S1**. Timeline for intervention design, delivery, and evaluation.

Patient Perceptions Toward Practice Components

Beginning approximately 12 weeks after the launch of the trial, we conducted semi-structured telephone interviews with 15 representative patients enrolled in the trial. Patient interviews assessed whether the intervention components, as designed and implemented, were perceived as appropriate, easy to access, easy to use, acceptable, desirable, credible, and useful in helping to achieve health-related goals.[4, 8] Transcripts of patient interviews were analyzed to identify and summarize perspectives raised regarding these important design attribute categories. Example themes and representative quotes were selected by group deliberation and consensus and are summarized in **Table S2** and discussed in the body of the accompanying manuscript. **Table S2** also includes potential implications for design refinements to improve implementation and effectiveness prior to a future larger scale trial.

**Table S2:** Patient perceptions regarding intervention components and their design implications.

| **Theme** | **Representative quote(s)** | **Potential design change**  **[design attribute category]*** |
| --- | --- | --- |
| **Trust and distrust in different sources of support** | *… XXXXX is a great hospital. This is a program that they’re offering to help you shape up, get healthy, live a longer life, and that it’s worth looking into.*  *…* *The only reason that I said okay was because it said XXXXX [health system]. And I would check on the scale and, you know, and everywhere to prove that it was XXXXX doing this, because I trust XXXXX.*  *… in the back of my head, the only, only reason that I said okay was because it said XXXXX [health system]. And I would check on the scale and, you know, and everywhere to prove that it was XXXXX doing this, because I trust XXXXX…*  *…but had it been anybody else, I would have never done it. Because I thought – what they said to me was, “They are going to get you in there,” and then they are gonna say, “Now you have to sign up for another year” – whatever.*  *I had seen my doctor not too long before, and we discussed my health, and whatever. And she never, ever mentioned that she gave my name to this thing. That was the confusing part. Because then – I mean, I’m very thankful for it, don’t take me wrong. It was – everything was confusing, and then having friends telling me, “It’s a scam”—all of it together… I couldn’t really prove to them that it wasn’t a scam.* | - Provide more information & reassurances upfront about the people and technology components; data uses; ensure there is a clear understanding of the components and how they are all ‘valid’ and coordinated by their health system [Appropriateness & Credibility] - Ensure the health system understands that patients value their support of these services and would like to see them improve and increase over time [Appropriateness] |
| **Weight loss attempts are often motivated by other health or social goals** | *Well, I thought about it as, “You need to do this. You keep gaining weight and making problems for yourself and it’s more medication.” And, you know, you just don’t want to do that anymore.*  *[My doctor] said she still wanted me to stay on the medication for a few more months, and then when I got this option I was like, “I know if I drop some more weight, it’s gonna really regulate my blood pressure.” I wanted to come off those meds.*  *…my goal is to make sure that I don’t have any more cardiac adventures in my life. And so, this program really helped point me to an awareness*  *I know I need to lose weight. I want to be fit for my elderhood and be a healthy grandma and prevent diseases, if I can.* | - Strengthen message content focused on weight loss from healthy eating and physical activity as a means to improve other health goals, such as to reduce the need for medications to treat high blood pressure [Appropriateness, Acceptability, and Usefulness] |
| **Critical importance of different sources of supportive accountability** | *So it wasn’t new information, but it was someone to be accountable to. And knowing that I had those next appointments was part of that accountability. So it made me stricter with myself.*  *I didn’t always weigh myself every day, but for the most part I did; that, I liked a lot because it kind of keeps you accountable.*  *It gave me a sense of accountability. I had to get on there [the scale] so somebody could look at it and see. And I remember the nurse calling me saying, “Hey, you did pretty good this week.” Or this happened, or that happened, so we’re looking at your average, so it’s like, okay, cool.*  *I think, in the beginning for me, I probably needed to meet with somebody every week, if just for 20 minutes*  *The first thing that was a really great surprise was seeing the dietitian. Those are the people that I saw, the dietitian and the coach who helped me with the physical exercise were just wonderful.* | - Better clarify the roles of different professionals and opportunities to engage in the different forms of professional support; offer and encourage regular engagement [Appropriateness, Credibility, and Usefulness] - Explore how to optimize frequency, timing, and duration of ‘human’ coaching and support while preserving feasibility and sustainability for the delivery system [Acceptability, Desirability, and Usefulness] |
| **Strengths and limits of automated messages** | *I like the messages. It’s kind of like a gentle reminder or, “How are you doing? What’s going on?” So, to me, it was there, it was a reminder.*  *I thought the weekly MyChart messages were encouraging. I actually looked forward to them, because I was like, yes, it logged my progress, or, oh shoot, it picked up that I messed up. But I did look forward to the messages.*  *I really liked the Monday messages. So, I would get a notification in the app and I also would get an email, I believe, just saying, “Hey, congratulations! You met your goal!” so, that’s always encouraging.*  *What would be great would be to have a different type of messaging when the weight’s going up instead of weight going down…* *the way I was perceiving them was a negative, oh, you’ve been naughty, and so my whole thing is if there’s a way to put a positive vent to it…* *it would have been really nice to hear some more positive motivating messages.*  *I don’t understand why it’s 10 weeks and then boom, no messages, nothing. Shouldn’t it be – all this be geared towards the whole time where I have the health club, instead of just, okay, “10 pounds in 10 weeks,” and then boom, you’re kind of done.* | - Messages should focus most on the outcomes of behaviors under a person’s immediate control (e.g., engagement in intensive coaching) [Acceptability, Desirability, and Usefulness] - Any message content that does reference weight loss progress should highlight longer trends that diminish effects of normal weight variability or ‘a few bad days’ [Acceptability and Desirability] - Messages sent to patients who are not making weight loss progress should provide immediate emotional support and acknowledge their efforts [Acceptability and Desirability] - Explore feasibility of more immediate and longer duration nurse or other human outreach for individualized coaching [Desirability and Usefulness] |
| **eScales (and self-weighing) should be framed as a means for health coaches to track and customize support, rather than a direct source of support** | *I got absolutely no use out of that scale. There was no immediate gratification that I could look at data, or go on a chart, or go on a website and say, "So, how am I doing? What's the progress? What's going on?”…* *It wasn't giving me anything. Right? I mean, someone else was getting two pieces of information, my weight and when I stood on the scale. But I wasn't. Right? I mean, what am I getting out of that?*  *I wasn't quite sure how the program was set up. I wasn't quite sure what was happening with the data. There was no breakdown. I was getting some sort of text messaging that was, "Oh, you checked in today?" And you know, "Well done." I didn't know where the other side of the building was, so to speak.*  *I wasn’t sure in the beginning that I could call her or ask her anything. I just thought she was like overseeing the program. I didn’t realize that she was really part of the team. So, maybe just some more clarification of what role the nurse plays in the whole program.* | - Improve upfront information uses of technology and data; frame self-weighing as a simple behavior that patients can control and as a daily reminder of behavioral goals & strategies [Appropriateness, Acceptability, Ease of Access, and Usefulness] - Provide clear descriptions for how daily self-weighing helps coordinate and customize support services [Appropriateness and Acceptability] - Explore possible avenues for more immediate and direct rewards after completing daily weights (i.e., earlier reinforcement by staff or IT) [Credibility, Desirability, and Usefulness] - Patients have varying interest in technology; encourage and enable use of other technologies (e.g., Fitbit; MyFitnessPal) without requiring them [Ease of Use and Desirability] |
| **Recognizing and overcoming failure expectations** | [what, if anything, made it difficult for you to start the program?] *…really, just my own motivation. And maybe a little bit of fear of, can I do this? Can I really lose weight…*  *If you have a fear of stepping on that scale, and you don’t want to see what’s on that – see that number pop up, that could be hard. For me it’s a challenge…I don’t want to get on there because I don’t want to see.*  *I can be on any kind of diet, and I’m not losing weight. And I was desperate.*  *But I still – I still didn’t lose weight. But that’s not her [the dietitian’s] fault at all. That has to do with something with me.*  *I think support is a lot. That’s what it is. And by just speaking to somebody, you get hope again, you get motivation again, you don’t feel defeated* | - Acknowledge and address possible failure experiences and expectations; consider the role of automated messages, website tools, and early and ongoing personnel support to assess and support patients with low self-efficacy perceptions [Appropriateness, Acceptability, and Usefulness] |
| **Attention to emotional support** | *… you know, a lot of people eat out of emotion and things like that. So, do I – maybe I… I don’t know. If there is somehow, some way of making – I don’t know, a support group…yeah, some kind of support, and that is not only “how many pounds did you lose today, how much rice did you eat?” Or whatever…because I think most overweight people are overweight because of emotions…you know, it’s not that they don’t know you should work out and eat less.*  *…there is a little bit more about motivation, and troubleshooting, and some individualized stuff, that is not just about the nutrition and the physical activity. It’s about what gets in the way for us to be able to get ourselves, because, so, with me, the relationship with food and exercise has to do with stress management, and emotions, and how we relate to food. That part, it wasn’t being attended to, and that’s part of the reason why I’m having trouble now, with keeping it up.* | - Acknowledge and validate emotional reactions to weight loss goals and attempts in messages and other written content (i.e., website; patient materials) [Acceptability and Usefulness] - Ensure that personnel engaging with patients are trained to identify and provide coaching about emotions and how to manage them to achieve goals [Appropriateness and Credibility] |
| **The pandemic was a profound barrier to weight loss attempts** | *…the daily routine has been disrupted. I'm sure everybody on the planet has put on more weight, because you're inside more, you're around food all the time…yeah, the "COVID 10." I believe I've put on like 10 pounds…*  *…now with all that we’re going through I’ve gone up in weight instead of down, but I have no doubt that if there were no COVID, I would have already gotten to the 195.* | - Offer services via multiple channels that appeal to different patients; ideally formats could be switched (i.e., to remote support) if access is disrupted [Appropriateness and Ease of Access] - Need for improved recognition of emotional shifts (i.e., fear, self-doubt, fatalism) and support for empowerment and self-efficacy in response to social influences [Usefulness] - There is need for a comparison group trial to evaluate the effectiveness of services relative to ‘usual care,’ as many people may actually be gaining weight [Usefulness] |

* Design Attribute Category definitions: Appropriateness (perceived fit, relevance, or compatibility within the setting or clinical context); Ease to Access (minimizing access barriers); Ease of Use (usability of technology or other resources); Acceptability (satisfactory in meeting perceived needs); Desirability (likable and enjoyable); Credibility (people and information viewed as valid and trusted); Usefulness (perception of added value; helpfulness in achieving personal goal(s))

Financial Sustainability of Intervention Components

For purposes of understanding sustainability beyond the trial period, input from health system leaders and study team members was used to estimate costs for each intervention ingredient, including personnel, supplies, contract services, and facility and administrative needs. Administrative data systems captured the number and type of contacts made between nurses and study participants. Staff members were interviewed to estimate the amount of time they spent supporting different intervention activities over 6 and 12 months. Hourly employment expenses (salary, fringe, and administrative overhead costs) were multiplied by the total time spent by each employee to estimate total personnel costs. Costs of eScales, community intervention program fees, and development costs for health IT components were determined from invoice amounts. Maintenance costs for IT support were estimated from interviews of IT staff members, who were asked to estimate hours per week spent supporting the intervention components. Estimated delivery costs for each intervention component are summarized in **Table S3**.

**Table S3:** Estimated delivery costs from the health care system perspective.

|  |  |  | **Offered BLS** | | | | | | |  | **Offered CLS** | | | | | | |  | **Overall** | | |
| --- | --- | --- | --- | --- | --- | --- | --- | --- | --- | --- | --- | --- | --- | --- | --- | --- | --- | --- | --- | --- | --- |
|  |  |  | **1^st^ Half Year** | | |  | **2^nd^ Half Year** | | |  | **1^st^ Half Year** | | |  | **2^nd^ Half Year** | | |  | **1^st^ Half Year** | **2^nd^ Half Year** | **Full Year** |
| **Intervention Component** | **Cost per Unit ($US)** |  | **Total Units*** | **Total Cost**  **($US)** | **Cost per Person**  **($US)** |  | **Total Units*** | **Total Cost**  **($US)** | **Cost per Person**  **($US)** |  | **Total Units*** | **Total Cost**  **($US)** | **Cost per Person**  **($US)** |  | **Total Units*** | **Total Cost**  **($US)** | **Cost per Person**  **($US)** |  | **Cost per Person**  **($US)** | **Cost per Person**  **($US)** | **Cost per Person**  **($US)** |
| **Personnel†** |  |  |  |  |  |  |  |  |  |  |  |  |  |  |  |  |  |  |  |  |  |
| Nurse Care Coordinator Coach | 69,139 |  | 0.031 | 1,083 | 28.50 |  | 0.000 | 0 | 0.00 |  | 0.079 | 2,737 | 65.16 |  | 0.000 | 0 | 0.00 |  | 47.75 | 0.00 | 47.75 |
| Nurse Manager | 127,607 |  | 0.006 | 379 | 9.97 |  | 0.000 | 0 | 0.00 |  | 0.007 | 419 | 9.97 |  | 0.000 | 0 | 0.00 |  | 9.97 | 0.00 | 9.97 |
| Computer/Epic Programmer | 128,868 |  | 0.005 | 353 | 9.29 |  | 0.000 | 0 | 0.00 |  | 0.006 | 390 | 9.29 |  | 0.000 | 0 | 0.00 |  | 9.29 | 0.00 | 9.29 |
| **Total Personnel** |  |  |  | **1,815** | **47.76** |  |  | **0** | **0.00** |  |  | **3,546** | **84.42** |  | **0.00** | **0** | **0.00** |  | **67.01** | **0.00** | **67.01** |
|  |  |  |  |  |  |  |  |  |  |  |  |  |  |  |  |  |  |  |  |  |  |
| **Materials & Supplies** |  |  |  |  |  |  |  |  |  |  |  |  |  |  |  |  |  |  |  |  |  |
| eScales | 80 |  | 38 | 3,040 | 80.00 |  | 0 | 0 | 0.00 |  | 42 | 3,360 | 80.00 |  | 0 | 0 | 0.00 |  | 80.00 | 0.00 | 80.00 |
| eScale Batteries^‡^ | 4 |  | 74 | 296 | 7.79 |  | 0 | 0 | 0.00 |  | 83 | 332 | 7.90 |  | 0 | 0 | 0.00 |  | 7.85 | 0.00 | 7.85 |
| Shipping | 8 |  | 38 | 304 | 8.00 |  | 0 | 0 | 0.00 |  | 42 | 336 | 8.00 |  | 0 | 0 | 0.00 |  | 8.00 | 0.00 | 8.00 |
| Contracted Facility-based Coaching^§^ | 750 |  | 9 | 3,375 | 88.82 |  | 9 | 3,375 | 88.82 |  | 13 | 4,875 | 116.07 |  | 13 | 4,875 | 116.07 |  | 103.13 | 103.13 | 206.25 |
| Contracted Virtual Coaching^§^ | 540 |  | 5 | 1,350 | 35.53 |  | 5 | 1,350 | 35.53 |  | 9 | 2,430 | 57.86 |  | 9 | 2,430 | 57.86 |  | 47.25 | 47.25 | 94.50 |
| **Total Materials & Supplies** |  |  |  | **8,365** | **220.13** |  |  | **4,725** | **124.34** |  |  | **11,333** | **269.83** |  |  | **7,305** | **173.93** |  | **246.23** | **150.38** | **396.60** |
|  |  |  |  |  |  |  |  |  |  |  |  |  |  |  |  |  |  |  |  |  |  |
| **Facility & Administrative^‖^** |  |  |  | **599** | **15.76** |  |  | **0** | **0.00** |  |  | **1,170** | **27.86** |  |  | **0** | **0.00** |  | **22.11** | **0.00** | **22.11** |
|  |  |  |  |  |  |  |  |  |  |  |  |  |  |  |  |  |  |  |  |  |  |
| **Total for All Components** |  |  |  | **10,779** | **283.66** |  |  | **4,725** | **124.34** |  |  | **16,049** | **382.12** |  |  | **7,305** | **173.93** |  | **335.35** | **150.38** | **485.72** |

* Units for personnel are number of full-time equivalents (i.e., the equivalent number of people working full time for 6 months) to provide the services to 38 study patients offered Basic Lifestyle Support and 42 study patients offered Customized Lifestyle Support. Units of contracted facility-based or virtual coaching reflect the numbers of study patients in each arm who engaged in those services, resulting in invoice payments to the partner fitness facility to provide the services for 12 months.

† Personnel categories were mapped to job classifications published by the U.S. Bureau of Labor Statistics, and unit costs represented national median salary costs as published in the May 2020 Occupational Employment and Wage Statistics (OEWS) Tables, found at: [www.bls.gov/oes/current/oes_nat.htm](http://www.bls.gov/oes/current/oes_nat.htm); each median salary was inflated by the partnering health system’s fringe rate cost of 22.37%.

‡ Batteries were provided with each scale and replaced after 3 months (excluding 3 patients who never initialized their scale)

§ Contracted payment amounts per person enrolled per 12 months

‖ Facility & administrative support costs for existing professionals were estimated as 33% of worker's salary costs

**Section 4: Patient Semi-Structured Interview Guide**

*Interviewer Intro: Hello this is [NAME, TITLE] calling from Northwestern University. You spoke with a member of our research team and scheduled this time for an interview about the [Health System Name] Connect4Health Program. This interview could take up to one hour. Is now still a good time to talk?*

***If NO:*** *Okay, a member of our research team will call you back to offer some different interview times. Thank you.*

***IF YES:*** *Okay, great. As a reminder, our research staff emailed you a copy of the consent form to review. Do you have any questions about consenting to participate in this study?*

*Patient Consented?*

*□ No*

*□ Yes*

***If No:*** *Interviewer: Thank you for your time. You will not be contacted about this specific study further.*

***If Yes****: Complete VERBAL CONSENT FORM and offer to email participant a signed copy.*

Introduction

*Interviewer:*

*You may recall that the [Site Name] Internal Medicine Practice site sent you a MyChart message in December or January, inviting you to take advantage of this program. The program included support to help you set a goal for healthy body weight and provided an electronic scale and additional information about programs that could help you become more physically active or eat healthy foods. We are evaluating this program with the goal of making it better.*

*Interviewer: Thank you for your willingness to participate in this interview today. As we discussed, we are interested in your perceptions and experiences about the [Health System Name] Connect4Health program that you participated in over the past few months. Anything you say today will be kept confidential. You are free to skip any questions that you don’t feel comfortable answering.*

**[AUDIOTAPE CONSENT]** *To ensure I capture everything that you tell me in your own words, I would like to audiotape this phone call. All audio records will be destroyed at end of the study.*

**Do you give me permission to audio tape this telephone conversation?**

*□*YES

*□*NO

***If Yes:*** Great, I'm going to turn on the audio recorder now. [TURN RECORDER ON]

***If No:*** OK, that's fine. We can still do the interview. I may pause every once in a while to make sure I can write down everything you are telling me.

*To start off today, I’d like to get an overall picture of your thoughts about the [Health System Name] Connect4Health Program.*

1. How would you describe the [Health System Name] Connect4Health program (e.g. to a family member/friend)?

PROBE (if necessary): That is, if someone asked you to describe the program to them, what would you say?

1. What motivated you to take part in this program?

PROBE (if necessary): What was the main reason you decided to take part in this program?

PROBE: What other reasons, if any, led you to decide to participate?

*Interviewer: Next I would like to get your thoughts about specific parts of the [Health System Name] Connect4Health Program. As a reminder, the Connect4Health program was designed to help [Site Name] Internal Medicine patients live healthier lifestyles and achieve healthy weight. The program involved several parts, including:*

- - *A digitally connected weight scale sent to you to use at home*
  - *A goal to lose 10 pounds in 10 weeks*
  - *Stepping on the scale every day*
  - *Free access to [Fitness Partner Org Name] and other diet and exercise resources.*
  - *IF PATIENT ASSIGNED TO BRIEF INTERVENTION ARM:*

*MyChart messages to help you achieve your weight loss goal*

*IF PATIENT ASSIGNED TO C3PO INTERVENTION ARM:*

*MyChart messages to help you achieve your weight loss goal and phone calls from nurses at the [Site Name] practice*

1. Which of these parts of the program did you like best and why?
2. Which parts of the program parts did you like least and why?

*Thank you. I know you just told me about the parts you liked the best and those you liked the least; I want to take a few minutes now to ask you about some of the other parts of the program that you didn’t mention.*

1. Can you tell me what you thought about [*Include all not addressed in Question 3 or 4*]:
   1. The digitally connected weight scale
   2. The goal to lose 10 pounds in 10 weeks
   3. Stepping on the scale every day
   4. Free access to [Fitness Partner Org Name] and other diet and exercise resources.
   5. IF PATIENT ASSIGNED TO BRIEF INTERVENTION ARM:

MyChart messages to help you achieve your weight loss goal

IF PATIENT ASSIGNED TO C3PO INTERVENTION ARM:

MyChart messages to help you achieve your weight loss goal and phone calls from nurses at the [Site Name] practice

*Thank you. The next set of questions is designed for us to learn whether or not you had any challenges or difficulties in participating in the [Health System Name] Connect4Health Program.*

1. What, if anything, made it difficult for you **to start** the program?

PROBE: How were you able to overcome that difficulty?

1. What, if anything, made it difficult for you to **stick with** the 10-week program? (*Interviewer note: If participant mentions the end of the program, no longer receiving messages, or COVID-19 related challenges, direct them to think back to their time during the 10-week program)*
2. Overall, what part (or parts) of the program did you find to be the most difficult for you?

PROBE (if necessary): Were there any other parts (i.e., setting up and using the scale, connecting to an intensive lifestyle intervention resource) that were difficult for you?

1. As part of this program, you were given three goals: Weighing yourself daily, losing 10 pounds in 10 weeks, and connecting to an intensive lifestyle intervention resource (for example, [Fitness Partner Org Name]). How did you feel about having these 3 goals set for you?

PROBE (if necessary): Did the goals fit well with your own healthy lifestyle goals?

1. What changes have you made to improve your health since beginning this program?

PROBE (if necessary): For example, physical activity, changes in your diet, or anything else.

1. Were there any changes that you tried to make and were unable to maintain? If so, why?

*My final few questions relate to any suggestions and feedback you may have about the program and how we might be able to improve it moving forward.*

1. What suggestions do you have to improve the program?

PROBE: In terms of the technology (for example the scale, MyChart messages, etc) is there anything you would change?

PROBE: In terms of the contact you received from the [Site Name] nurses, is there anything you would change?

1. What parts of the program would you keep exactly as is? Why?
2. Would you recommend the *[Health System Name] Connect4Health* program to a family member or friend? Why or why not?
3. Before we end, is there anything else you would like to share with me about your participating in the [Health System Name] Connect4Health Program?

*Interviewer:* *Thank you for your time, your input is very valuable to this research project. As a thank you for your participation, we will be sending you a $75 gift card within two weeks. Can you please confirm your mailing address?*

Address: ____________________________________________________________________

*Thank you again. If you have any additional questions about your participation in this study, please reference the consent form you were emailed for contact information for the research team and the Northwestern University Institutional Review Board.*

[TURN RECORDER OFF]

**References for Multimedia Appendix**

[1] E. S. LeBlanc, C. D. Patnode, E. M. Webber, N. Redmond, M. Rushkin, and E. A. O'Connor, "Behavioral and Pharmacotherapy Weight Loss Interventions to Prevent Obesity-Related Morbidity and Mortality in Adults: Updated Evidence Report and Systematic Review for the US Preventive Services Task Force," *JAMA,* vol. 320, no. 11, pp. 1172-1191, Sep 18 2018, doi: 10.1001/jama.2018.7777. PubMed PMID: 30326501.

[2] E. A. O'Connor, C. V. Evans, M. C. Rushkin, N. Redmond, and J. S. Lin, *Behavioral counseling interventions to promote a healthy diet and physical activity for cardiovascular disease prevention in adults with cardiovascular risk factors: updated systematic review for the U.S. preventive services task force*, Rockville (MD): Agency for Healthcare Research and Quality (US),, 2020, p. 1 online resource (1 PDF file (x pages)). [Online]. Available: <http://www.ncbi.nlm.nih.gov/books/NBK565468/>.

[3] E. M. Venditti and M. K. Kramer, "Necessary components for lifestyle modification interventions to reduce diabetes risk," *Curr Diab Rep,* vol. 12, no. 2, pp. 138-46, Apr 2012, doi: 10.1007/s11892-012-0256-9. PubMed PMID: 22350807.

[4] A. R. Lyon and K. Koerner, "User-Centered Design for Psychosocial Intervention Development and Implementation," *Clin Psychol (New York),* vol. 23, no. 2, pp. 180-200, Jun 2016, doi: 10.1111/cpsp.12154. PubMed PMID: 29456295; PMCID: PMC5812700.

[5] Y. Zheng, M. L. Klem, S. M. Sereika, C. A. Danford, L. J. Ewing, and L. E. Burke, "Self-weighing in weight management: a systematic literature review," *Obesity (Silver Spring),* vol. 23, no. 2, pp. 256-65, Feb 2015, doi: 10.1002/oby.20946. PubMed PMID: 25521523.

[6] C. D. Madigan, A. J. Daley, A. L. Lewis, P. Aveyard, and K. Jolly, "Is self-weighing an effective tool for weight loss: a systematic literature review and meta-analysis," (in eng), *The international journal of behavioral nutrition and physical activity,* vol. 12, p. 104, Aug 21 2015, doi: 10.1186/s12966-015-0267-4. PubMed PMID: 26293454; PMCID: PMC4546162.

[7] I. BodyTrace. "BodyTrace eScale." <https://www.bodytrace.com/medical/> (accessed May 18, 2022).

[8] B. J. Weiner *et al.*, "Psychometric assessment of three newly developed implementation outcome measures," *Implement Sci,* vol. 12, no. 1, p. 108, Aug 29 2017, doi: 10.1186/s13012-017-0635-3. PubMed PMID: 28851459; PMCID: PMC5576104.
