# Supplementary material for: Hybrid Health IT and Telehealth–Delivered Behavioral Weight Loss Services for Primary Care Patients With Cardiovascular Risk Factors: Intervention Component Design and Pragmatic Randomized Feasibility Trial
Source: JMIR Mhealth Uhealth. 2025 Oct 22;13:e58722. doi: 10.2196/58722 (PMC12543212; doi:10.2196/58722)
Supplement: Checklist 1 [file mhealth-v13-e58722-s002.pdf]

CONSORT-EHEALTH (V 1.6.1) - Submission/Publication Form

The CONSORT-EHEALTH checklist is intended for authors of randomized trials evaluating web-based and Internet-based applications/interventions, including mobile interventions, electronic games (incl multiplayer games), social media, certain telehealth applications, and other interactive and/or networked electronic applications. Some of the items (e.g. all subitems under item 5 - description of the intervention) may also be applicable for other study designs.

The goal of the CONSORT EHEALTH checklist and guideline is to be
a) a guide for reporting for authors of RCTs,
b) to form a basis for appraisal of an ehealth trial (in terms of validity)

CONSORT-EHEALTH items/subitems are MANDATORY reporting items for studies published in the Journal of Medical Internet Research and other journals / scientific societies endorsing the checklist.

Items numbered 1., 2., 3., 4a., 4b etc are original CONSORT or CONSORT-NPT (non-pharmacologic treatment) items.
Items with Roman numerals (i., ii, iii, iv etc.) are CONSORT-EHEALTH extensions/clarifications.

As the CONSORT-EHEALTH checklist is still considered in a formative stage, we would ask that you also RATE ON A SCALE OF 1-5 how important/useful you feel each item is FOR THE PURPOSE OF THE CHECKLIST and reporting guideline (optional).

Mandatory reporting items are marked with a red *.
In the textboxes, either copy & paste the relevant sections from your manuscript into this form - please include any quotes from your manuscript in QUOTATION MARKS,
or answer directly by providing additional information not in the manuscript, or elaborating on why the item was not relevant for this study.

YOUR ANSWERS WILL BE PUBLISHED AS A SUPPLEMENTARY FILE TO YOUR PUBLICATION IN JMIR AND ARE CONSIDERED PART OF YOUR PUBLICATION (IF ACCEPTED).
Please fill in these questions diligently. Information will not be copyedited, so please use proper spelling and grammar, use correct capitalization, and avoid abbreviations.

DO NOT FORGET TO SAVE AS PDF _AND_ CLICK THE SUBMIT BUTTON SO YOUR ANSWERS ARE IN OUR DATABASE !!!

Citation Suggestion (if you append the pdf as Appendix we suggest to cite this paper in the caption):
Eysenbach G, CONSORT-EHEALTH Group

CONSORT-EHEALTH: Improving and Standardizing Evaluation Reports of Web-based and Mobile Health Interventions
J Med Internet Res 2011;13(4):e126
URL: http://www.jmir.org/2011/4/e126/
doi: 10.2196/jmir.1923
PMID: 22209829

Sign in to Google to save your progress. Learn more

* Indicates required question

Your name *

First Last

Ronald Ackermann

Primary Affiliation (short), City, Country *

University of Toronto, Toronto, Canada

Northwestern University, Chicago, IL, USA

Your e-mail address *

abc@gmail.com

r.ackermann@northwestern.edu

Title of your manuscript *

Provide the (draft) title of your manuscript.

Hybrid Health IT Plus Telehealth Delivery of Behavioral Weight Loss Services for Adult Primary Care Patients with Cardiovascular Risk Factors: Design of Intervention Components and Results of a Pragmatic Randomized Feasibility Trial

Name of your App/Software/Intervention *

If there is a short and a long/alternate name, write the short name first and add the long name in brackets.

NM Connect, a.k.a., C3PO [Coordinated Pragm

Evaluated Version (if any)

e.g. "V1", "Release 2017-03-01", "Version 2.0.27913"

Your answer

Language(s) *

What language is the intervention/app in? If multiple languages are available, separate by comma (e.g. "English, French")

English

URL of your Intervention Website or App

e.g. a direct link to the mobile app on app in appstore (itunes, Google Play), or URL of the website. If the intervention is a DVD or hardware, you can also link to an Amazon page.

Your answer

URL of an image/screenshot (optional)

Your answer

Accessibility *

Can an enduser access the intervention presently?

- ( ) access is free and open
- ( ) access only for special usergroups, not open
- ( ) access is open to everyone, but requires payment/subscription/in-app purchases
- ( ) app/intervention no longer accessible
- (x) Other: Our study evaluates a prototype prior to larger scale testing; the interv

Primary Medical Indication/Disease/Condition *

e.g. "Stress", "Diabetes", or define the target group in brackets after the condition, e.g. "Autism (Parents of children with)", "Alzheimers (Informal Caregivers of)"

Overweight and Obesity with one or more card

Primary Outcomes measured in trial *

comma-separated list of primary outcomes reported in the trial

preliminary weight loss pilot data

Secondary/other outcomes

Are there any other outcomes the intervention is expected to affect?

feasibility of trial procedures, intervention feasibility, acceptability, desirability, and usability

Recommended "Dose" *

What do the instructions for users say on how often the app should be used?

- ( ) Approximately Daily
- (•) Approximately Weekly
- ( ) Approximately Monthly
- ( ) Approximately Yearly
- ( ) "as needed"
- ( ) Other:

Approx. Percentage of Users (starters) still using the app as recommended after 3 months *

- (•) unknown / not evaluated
- ( ) 0-10%
- ( ) 11-20%
- ( ) 21-30%
- ( ) 31-40%
- ( ) 41-50%
- ( ) 51-60%
- ( ) 61-70%
- ( ) 71%-80%
- ( ) 81-90%
- ( ) 91-100%
- ( ) Other:

Overall, was the app/intervention effective? *

- ○ yes: all primary outcomes were significantly better in intervention group vs control
- ○ partly: SOME primary outcomes were significantly better in intervention group vs control
- ○ no statistically significant difference between control and intervention
- ○ potentially harmful: control was significantly better than intervention in one or more outcomes
- ◉ inconclusive: more research is needed
- ○ Other:

Article Preparation Status/Stage *

At which stage in your article preparation are you currently (at the time you fill in this form)

- ○ not submitted yet - in early draft status
- ○ not submitted yet - in late draft status, just before submission
- ○ submitted to a journal but not reviewed yet
- ◉ submitted to a journal and after receiving initial reviewer comments
- ○ submitted to a journal and accepted, but not published yet
- ○ published
- ○ Other:

Journal *

If you already know where you will submit this paper (or if it is already submitted), please provide the journal name (if it is not JMIR, provide the journal name under "other")

- ○ not submitted yet / unclear where I will submit this
- ○ Journal of Medical Internet Research (JMIR)
- ● JMIR mHealth and UHealth
- ○ JMIR Serious Games
- ○ JMIR Mental Health
- ○ JMIR Public Health
- ○ JMIR Formative Research
- ○ Other JMIR sister journal
- ○ Other:

Is this a full powered effectiveness trial or a pilot/feasibility trial? *

- ● Pilot/feasibility
- ○ Fully powered

Manuscript tracking number *

If this is a JMIR submission, please provide the manuscript tracking number under "other" (The ms tracking number can be found in the submission acknowledgement email, or when you login as author in JMIR. If the paper is already published in JMIR, then the ms tracking number is the four-digit number at the end of the DOI, to be found at the bottom of each published article in JMIR)

- ○ no ms number (yet) / not (yet) submitted to / published in JMIR
- ● Other: JMU ms#58722

TITLE AND ABSTRACT

1a) TITLE: Identification as a randomized trial in the title

1a) Does your paper address CONSORT item 1a? *

I.e does the title contain the phrase "Randomized Controlled Trial"? (if not, explain the reason under "other")

- (selected) yes
- Other:

1a-i) Identify the mode of delivery in the title

Identify the mode of delivery. Preferably use “web-based” and/or “mobile” and/or “electronic game” in the title. Avoid ambiguous terms like “online”, “virtual”, “interactive”. Use “Internet-based” only if Intervention includes non-web-based Internet components (e.g. email), use “computer-based” or “electronic” only if offline products are used. Use “virtual” only in the context of “virtual reality” (3-D worlds). Use “online” only in the context of “online support groups”. Complement or substitute product names with broader terms for the class of products (such as “mobile” or “smart phone” instead of “iphone”), especially if the application runs on different platforms.

	1	2	3	4	5	
subitem not at all important	○	○	●	○	○	essential

Clear selection

Does your paper address subitem 1a-i? *

Copy and paste relevant sections from manuscript title (include quotes in quotation marks "like this" to indicate direct quotes from your manuscript), or elaborate on this item by providing additional information not in the ms, or briefly explain why the item is not applicable/relevant for your study

The intervention has multiple components, including IT coaching tools accessed via the web or an app, as well as telephone delivery. Described like this: "Hybrid Health IT Plus Telehealth Delivery of Behavioral Weight Loss Services..."

1a-ii) Non-web-based components or important co-interventions in title

Mention non-web-based components or important co-interventions in title, if any (e.g., “with telephone support”).

	1	2	3	4	5	
subitem not at all important	○	◉	○	○	○	essential

Clear selection

Does your paper address subitem 1a-ii?

Copy and paste relevant sections from manuscript title (include quotes in quotation marks "like this" to indicate direct quotes from your manuscript), or elaborate on this item by providing additional information not in the ms, or briefly explain why the item is not applicable/relevant for your study

The intervention has multiple components, including IT coaching tools accessed via the web or an app, as well as telephone delivery. Described like this: "Hybrid Health IT Plus Telehealth Delivery of Behavioral Weight Loss Services..."

1a-iii) Primary condition or target group in the title

Mention primary condition or target group in the title, if any (e.g., “for children with Type I Diabetes”) Example: A Web-based and Mobile Intervention with Telephone Support for Children with Type I Diabetes: Randomized Controlled Trial

	1	2	3	4	5	
subitem not at all important	○	○	◉	○	○	essential

Clear selection

Does your paper address subitem 1a-iii? *

Copy and paste relevant sections from manuscript title (include quotes in quotation marks "like this" to indicate direct quotes from your manuscript), or elaborate on this item by providing additional information not in the ms, or briefly explain why the item is not applicable/relevant for your study

Like this: "...for Adult Primary Care Patients with Cardiovascular Risk Factors..."

1b) ABSTRACT: Structured summary of trial design, methods, results, and conclusions

NPT extension: Description of experimental treatment, comparator, care providers, centers, and blinding status.

1b-i) Key features/functionalities/components of the intervention and comparator in the METHODS section of the ABSTRACT

Mention key features/functionalities/components of the intervention and comparator in the abstract. If possible, also mention theories and principles used for designing the site. Keep in mind the needs of systematic reviewers and indexers by including important synonyms. (Note: Only report in the abstract what the main paper is reporting. If this information is missing from the main body of text, consider adding it)

	1	2	3	4	5	
subitem not at all important	○	○	●	○	○	essential

Clear selection

Does your paper address subitem 1b-i? *

Copy and paste relevant sections from the manuscript abstract (include quotes in quotation marks "like this" to indicate direct quotes from your manuscript), or elaborate on this item by providing additional information not in the ms, or briefly explain why the item is not applicable/relevant for your study

Like this: "...offering “Basic Lifestyle Services” (BLS), which provided a scale that transmits weight data to the EHR using cellular networks, a coupon to enroll in lifestyle coaching resources through a partnering fitness organization, and periodic EHR messages..."

1b-ii) Level of human involvement in the METHODS section of the ABSTRACT

Clarify the level of human involvement in the abstract, e.g., use phrases like “fully automated” vs. “therapist/nurse/care provider/physician-assisted” (mention number and expertise of providers involved, if any). (Note: Only report in the abstract what the main paper is reporting. If this information is missing from the main body of text, consider adding it)

	1	2	3	4	5	
subitem not at all important	○	○	●	○	○	essential

Clear selection

Does your paper address subitem 1b-ii?

Copy and paste relevant sections from the manuscript abstract (include quotes in quotation marks "like this" to indicate direct quotes from your manuscript), or elaborate on this item by providing additional information not in the ms, or briefly explain why the item is not applicable/relevant for your study

Like this: "...including weekly email messages adaptive to weight loss progress and telephonic coaching by a nurse for those facing challenges..."

1b-iii) Open vs. closed, web-based (self-assessment) vs. face-to-face assessments in the METHODS section of the ABSTRACT

Mention how participants were recruited (online vs. offline), e.g., from an open access website or from a clinic or a closed online user group (closed usergroup trial), and clarify if this was a purely web-based trial, or there were face-to-face components (as part of the intervention or for assessment). Clearly say if outcomes were self-assessed through questionnaires (as common in web-based trials). Note: In traditional offline trials, an open trial (open-label trial) is a type of clinical trial in which both the researchers and participants know which treatment is being administered. To avoid confusion, use “blinded” or “unblinded” to indicated the level of blinding instead of “open”, as “open” in web-based trials usually refers to “open access” (i.e. participants can self-enrol). (Note: Only report in the abstract what the main paper is reporting. If this information is missing from the main body of text, consider adding it)

	1	2	3	4	5	
subitem not at all important	○	○	◉	○	○	essential

Clear selection

Does your paper address subitem 1b-iii?

Copy and paste relevant sections from the manuscript abstract (include quotes in quotation marks "like this" to indicate direct quotes from your manuscript), or elaborate on this item by providing additional information not in the ms, or briefly explain why the item is not applicable/relevant for your study

All participants were recruited from the active patient registry of a partnering health system, described like this: "...Patients with BMI ≥27 and ≥1 cardiovascular risk factor were sent a single electronic health record (EHR) message between December 2019 and January 2020 offering services to support a weight loss goal of 10 pounds in 10 weeks. All patients who affirmed interest were pragmatically enrolled..." There was no contact with research staff and recruitment followed an IRB approved waiver of written informed consent. All people who opted in to receive the interventions from the health system were included in the trial.

1b-iv) RESULTS section in abstract must contain use data

Report number of participants enrolled/assessed in each group, the use/uptake of the intervention (e.g., attrition/adherence metrics, use over time, number of logins etc.), in addition to primary/secondary outcomes. (Note: Only report in the abstract what the main paper is reporting. If this information is missing from the main body of text, consider adding it)

	1	2	3	4	5	
subitem not at all important	○	○	◉	○	○	essential

Clear selection

Does your paper address subitem 1b-iv?

Copy and paste relevant sections from the manuscript abstract (include quotes in quotation marks "like this" to indicate direct quotes from your manuscript), or elaborate on this item by providing additional information not in the ms, or briefly explain why the item is not applicable/relevant for your study

Like this: "...and 80 (18.8%) affirmed interest in the weight loss goal and were enrolled..." and this: "...About half (n=42) of participants were randomized by an EHR algorithm to also receive..." and like this: "18 (43%) CLS patients and 8 (21%) BLS patients performed daily self-weighing (P=.06), and 22 (52%) CLS patients and 14 (37%) BLS patients enrolled in referral-based lifestyle resources (P=.18)."

1b-v) CONCLUSIONS/DISCUSSION in abstract for negative trials

Conclusions/Discussions in abstract for negative trials: Discuss the primary outcome - if the trial is negative (primary outcome not changed), and the intervention was not used, discuss whether negative results are attributable to lack of uptake and discuss reasons. (Note: Only report in the abstract what the main paper is reporting. If this information is missing from the main body of text, consider adding it)

	1	2	3	4	5	
subitem not at all important	○	●	○	○	○	essential

Clear selection

Does your paper address subitem 1b-v?

Copy and paste relevant sections from the manuscript abstract (include quotes in quotation marks "like this" to indicate direct quotes from your manuscript), or elaborate on this item by providing additional information not in the ms, or briefly explain why the item is not applicable/relevant for your study

The goal of demonstrating feasibility was established so in that sense the trial was not "negative." The need for additional definitive research is described like this: "...procedures proved feasible and interventions showed preliminary effectiveness warranting further study in a larger trial..."

INTRODUCTION

2a) In INTRODUCTION: Scientific background and explanation of rationale

2a-i) Problem and the type of system/solution

Describe the problem and the type of system/solution that is object of the study: intended as stand-alone intervention vs. incorporated in broader health care program? Intended for a particular patient population? Goals of the intervention, e.g., being more cost-effective to other interventions, replace or complement other solutions? (Note: Details about the intervention are provided in "Methods" under 5)

	1	2	3	4	5	
subitem not at all important	○	○	●	○	○	essential

Clear selection

Does your paper address subitem 2a-i? *

Copy and paste relevant sections from the manuscript (include quotes in quotation marks "like this" to indicate direct quotes from your manuscript), or elaborate on this item by providing additional information not in the ms, or briefly explain why the item is not applicable/relevant for your study

Like this: "There is an immense need for research that identifies feasible and acceptable strategies for health systems to implement ILI."

2a-ii) Scientific background, rationale: What is known about the (type of) system

Scientific background, rationale: What is known about the (type of) system that is the object of the study (be sure to discuss the use of similar systems for other conditions/diagnoses, if appropiate), motivation for the study, i.e. what are the reasons for and what is the context for this specific study, from which stakeholder viewpoint is the study performed, potential impact of findings [2]. Briefly justify the choice of the comparator.

	1	2	3	4	5	
subitem not at all important	○	○	●	○	○	essential

Clear selection

Does your paper address subitem 2a-ii? *

Copy and paste relevant sections from the manuscript (include quotes in quotation marks "like this" to indicate direct quotes from your manuscript), or elaborate on this item by providing additional information not in the ms, or briefly explain why the item is not applicable/relevant for your study

Like this: "...health systems face challenges implementing ILI,[11, 12] which require dedicated personnel, space, supplies, and technologies that generally do not align well with the existing structure, workforce, and routines of healthcare delivery..." and this: "...Despite increasing numbers of ILI programs nationally, clinicians refer very few patients to ILI, and most people who may benefit remain unaware.[19] There is an immense need for research that identifies feasible and acceptable strategies for health systems to implement ILI...."

2b) In INTRODUCTION: Specific objectives or hypotheses

Does your paper address CONSORT subitem 2b? *

Copy and paste relevant sections from the manuscript (include quotes in quotation marks "like this" to indicate direct quotes from your manuscript), or elaborate on this item by providing additional information not in the ms, or briefly explain why the item is not applicable/relevant for your study

Like this: "The aim of this paper is to describe the collaborative approach used for creation of the ILI implementation strategies and to report preliminary data for the feasibility, acceptability, and potential for effectiveness of this approach."

METHODS

3a) Description of trial design (such as parallel, factorial) including allocation ratio

Does your paper address CONSORT subitem 3a? *

Copy and paste relevant sections from the manuscript (include quotes in quotation marks "like this" to indicate direct quotes from your manuscript), or elaborate on this item by providing additional information not in the ms, or briefly explain why the item is not applicable/relevant for your study

Like this: "...and a small parallel, 2-arm, pilot trial with 1:1 individual treatment assignment..."

3b) Important changes to methods after trial commencement (such as eligibility criteria), with reasons

Does your paper address CONSORT subitem 3b? *

Copy and paste relevant sections from the manuscript (include quotes in quotation marks "like this" to indicate direct quotes from your manuscript), or elaborate on this item by providing additional information not in the ms, or briefly explain why the item is not applicable/relevant for your study

No major changes were made to the protocol after trial commencement

3b-i) Bug fixes, Downtimes, Content Changes

Bug fixes, Downtimes, Content Changes: ehealth systems are often dynamic systems. A description of changes to methods therefore also includes important changes made on the intervention or comparator during the trial (e.g., major bug fixes or changes in the functionality or content) (5-iii) and other "unexpected events" that may have influenced study design such as staff changes, system failures/downtimes, etc. [2].

	1	2	3	4	5	
subitem not at all important	○	◉	○	○	○	essential

Clear selection

Does your paper address subitem 3b-i?

Copy and paste relevant sections from the manuscript (include quotes in quotation marks "like this" to indicate direct quotes from your manuscript), or elaborate on this item by providing additional information not in the ms, or briefly explain why the item is not applicable/relevant for your study

The feasibility trial was short. Although much was learned to improve the interventions after completion of the trial, no bug fixes or downtime issues were encountered during the active trial period.

4a) Eligibility criteria for participants

Does your paper address CONSORT subitem 4a? *

Copy and paste relevant sections from the manuscript (include quotes in quotation marks "like this" to indicate direct quotes from your manuscript), or elaborate on this item by providing additional information not in the ms, or briefly explain why the item is not applicable/relevant for your study

Like this: "...Inclusion criteria were: age 18-75 years; ≥1 log-in within the past 6 months to MyChart (i.e., a secure EHR access portal for patients to view their chart and exchange information with care team members); completion of ≥1 office visit in the past 6 months at the participating primary care practice site; measured weight and height at the most recent office visit indicating body mass index (BMI) ≥27.0 kg/m2; and ≥1 cardiometabolic risk factor (high blood pressure; abnormal blood cholesterol, prediabetes, or type 2 diabetes). Patients were ineligible if their last blood pressure was ≥180/105 mmHg or last hemoglobin A1c was ≥10.9%. Patients were also ineligible if they received cancer treatment in the past 2 years, were hospitalized in the past 3 months, were pregnant, or had personal history of an eating disorder or serious mental illness."

4a-i) Computer / Internet literacy

Computer / Internet literacy is often an implicit “de facto” eligibility criterion - this should be explicitly clarified.

	1	2	3	4	5	
subitem not at all important	○	○	○	●	○	essential

Clear selection

Does your paper address subitem 4a-i?

Copy and paste relevant sections from the manuscript (include quotes in quotation marks "like this" to indicate direct quotes from your manuscript), or elaborate on this item by providing additional information not in the ms, or briefly explain why the item is not applicable/relevant for your study

Like this: "Inclusion criteria were: age 18-75 years; ≥1 log-in within the past 6 months to MyChart (i.e., a secure EHR access portal for patients to view their chart and exchange information with care team members); completion of ≥1 office visit in the past 6 months at the participating primary care practice site; measured weight and height at the most recent office visit indicating body mass index (BMI) ≥27.0 kg/m2; and ≥1 cardiometabolic risk factor (high blood pressure; abnormal blood cholesterol, prediabetes, or type 2 diabetes). Patients were ineligible if their last blood pressure was ≥180/105 mmHg or last hemoglobin A1c was ≥10.9%. Patients were also ineligible if they received cancer treatment in the past 2 years, were hospitalized in the past 3 months, were pregnant, or had personal history of an eating disorder or serious mental illness..."

4a-ii) Open vs. closed, web-based vs. face-to-face assessments:

Open vs. closed, web-based vs. face-to-face assessments: Mention how participants were recruited (online vs. offline), e.g., from an open access website or from a clinic, and clarify if this was a purely web-based trial, or there were face-to-face components (as part of the intervention or for assessment), i.e., to what degree got the study team to know the participant. In online-only trials, clarify if participants were quasi-anonymous and whether having multiple identities was possible or whether technical or logistical measures (e.g., cookies, email confirmation, phone calls) were used to detect/prevent these.

	1	2	3	4	5	
subitem not at all important	○	○	○	●	○	essential

Clear selection

Does your paper address subitem 4a-ii? *

Copy and paste relevant sections from the manuscript (include quotes in quotation marks "like this" to indicate direct quotes from your manuscript), or elaborate on this item by providing additional information not in the ms, or briefly explain why the item is not applicable/relevant for your study

Recruitment was offline, within a single clinical delivery system, described like this: "...the data manager used the EHR's batch patient messaging tool to transmit a standardized outreach “goal setting” message to each patient remaining on the list. The message encouraged healthy eating and physical activity changes with a weight loss goal, and it offered free-of-charge access to technologies and coaching support to help recipients achieve the goal. The message also prompted patients to click an embedded link if they wished to adopt a weight loss goal and receive support. Because limited resources were available to support intervention costs during feasibility testing, we sent MyChart messages to only 100 - 200 patients every 2 weeks until the target sample size of 80 patients had affirmed interest in receiving the intervention services..."

4a-iii) Information giving during recruitment

Information given during recruitment. Specify how participants were briefed for recruitment and in the informed consent procedures (e.g., publish the informed consent documentation as appendix, see also item X26), as this information may have an effect on user self-selection, user expectation and may also bias results.

	1	2	3	4	5	
subitem not at all important	(•)	○	○	○	○	essential

Clear selection

Does your paper address subitem 4a-iii?

Copy and paste relevant sections from the manuscript (include quotes in quotation marks "like this" to indicate direct quotes from your manuscript), or elaborate on this item by providing additional information not in the ms, or briefly explain why the item is not applicable/relevant for your study

Our study enrolled all individuals who engaged in new health promotion services offered by their healthcare team. The study enrolled and randomized patients under an IRB-approved waiver of written informed consent. Research procedures were not described to patients. Described like this: "...The trial involved pragmatic recruitment, enrollment, and data collection strategies.[27] Eligibility determination and intervention allocation were automated within the EHR, trial enrollment proceeded with an IRB-approved waiver of written informed consent, and most outcomes were ascertained from administrative data systems. Each of these approaches minimized contact between study patients and the research team and enabled study patients and the interventions they received to approximate “real world” conditions."

4b) Settings and locations where the data were collected

Does your paper address CONSORT subitem 4b? *

Copy and paste relevant sections from the manuscript (include quotes in quotation marks "like this" to indicate direct quotes from your manuscript), or elaborate on this item by providing additional information not in the ms, or briefly explain why the item is not applicable/relevant for your study

Like this: "in a single general internal medicine practice setting in downtown Chicago, Illinois, USA."

4b-i) Report if outcomes were (self-)assessed through online questionnaires

Clearly report if outcomes were (self-)assessed through online questionnaires (as common in web-based trials) or otherwise.

	1	2	3	4	5	
subitem not at all important	○	○	◉	○	○	essential

Clear selection

Does your paper address subitem 4b-i? *

Copy and paste relevant sections from the manuscript (include quotes in quotation marks "like this" to indicate direct quotes from your manuscript), or elaborate on this item by providing additional information not in the ms, or briefly explain why the item is not applicable/relevant for your study

Outcome data for the trial were collected from clinical and administrative systems with no direct contact with members of the research team. A random subset of trial participants was recruited for semi-structured interviews. Data collection is described like this: "and most outcomes were ascertained from administrative data systems..." and this: "...Patient reported data were collected by semi-structured telephone interviews with 15 randomly-selected patients."

4b-ii) Report how institutional affiliations are displayed

Report how institutional affiliations are displayed to potential participants [on ehealth media], as affiliations with prestigious hospitals or universities may affect volunteer rates, use, and reactions with regards to an intervention.(Not a required item – describe only if this may bias results)

	1	2	3	4	5	
subitem not at all important	○	○	◉	○	○	essential

Clear selection

Does your paper address subitem 4b-ii?

Copy and paste relevant sections from the manuscript (include quotes in quotation marks "like this" to indicate direct quotes from your manuscript), or elaborate on this item by providing additional information not in the ms, or briefly explain why the item is not applicable/relevant for your study

All study participants were patients of a single health system. They were enrolled in the research pragmatically after they affirmed interest in engaging with specific health promotion services offered by their healthcare team. All patients were familiar with their health system and healthcare team. They were not approached for research by the research team.

5) The interventions for each group with sufficient details to allow replication, including how and when they were actually administered

5-i) Mention names, credential, affiliations of the developers, sponsors, and owners

Mention names, credential, affiliations of the developers, sponsors, and owners [6] (if authors/evaluators are owners or developer of the software, this needs to be declared in a "Conflict of interest" section or mentioned elsewhere in the manuscript).

	1	2	3	4	5	
subitem not at all important	(selected)	○	○	○	○	essential

Clear selection

Does your paper address subitem 5-i?

Copy and paste relevant sections from the manuscript (include quotes in quotation marks "like this" to indicate direct quotes from your manuscript), or elaborate on this item by providing additional information not in the ms, or briefly explain why the item is not applicable/relevant for your study

There are no specific developers or business interests related to this intervention, which is currently still only available to select patients for research purposes. The manuscript does acknowledge the sources of support, like this: "...We would like to acknowledge Northwestern Medical Group Primary Care, Northwestern Memorial Healthcare, and the Fitness Formula Clubs for collaborating to develop and implement the practice interventions evaluated by this research..."

5-ii) Describe the history/development process

Describe the history/development process of the application and previous formative evaluations (e.g., focus groups, usability testing), as these will have an impact on adoption/use rates and help with interpreting results.

	1	2	3	4	5	
subitem not at all important	○	○	○	○	●	essential

Clear selection

Does your paper address subitem 5-ii?

Copy and paste relevant sections from the manuscript (include quotes in quotation marks "like this" to indicate direct quotes from your manuscript), or elaborate on this item by providing additional information not in the ms, or briefly explain why the item is not applicable/relevant for your study

This study is almost entirely about the development process, with preliminary tests of feasibility and usability reported throughout the main body of the paper and the supplemental online appendix. The manuscript reports these activities like this: "...Design goals were to cocreate ILI intervention components and implementation strategies that are evidence-based,[1, 2] and perceived by stakeholders as technically feasible, acceptable, and financially sustainable.[29] This approach followed collaborative design principles,[11] and produced ILI implementation strategies for support of weight loss goal setting, self-weighing, and recurring coaching support. Stakeholders advised using..."

5-iii) Revisions and updating

Revisions and updating. Clearly mention the date and/or version number of the application/intervention (and comparator, if applicable) evaluated, or describe whether the intervention underwent major changes during the evaluation process, or whether the development and/or content was “frozen” during the trial. Describe dynamic components such as news feeds or changing content which may have an impact on the replicability of the intervention (for unexpected events see item 3b).

	1	2	3	4	5	
subitem not at all important	○	○	◉	○	○	essential

Clear selection

Does your paper address subitem 5-iii?

Copy and paste relevant sections from the manuscript (include quotes in quotation marks "like this" to indicate direct quotes from your manuscript), or elaborate on this item by providing additional information not in the ms, or briefly explain why the item is not applicable/relevant for your study

Described like this: "...The experimental services were offered to patients beginning January 2020, and access to some services (details below) were interrupted by the coronavirus pandemic stay-at-home order in March 2020..."

5-iv) Quality assurance methods

Provide information on quality assurance methods to ensure accuracy and quality of information provided [1], if applicable.

	1	2	3	4	5	
subitem not at all important	○	◉	○	○	○	essential

Clear selection

Does your paper address subitem 5-iv?

Copy and paste relevant sections from the manuscript (include quotes in quotation marks "like this" to indicate direct quotes from your manuscript), or elaborate on this item by providing additional information not in the ms, or briefly explain why the item is not applicable/relevant for your study

The entire manuscript has extensive details regarding data collection and analysis. Further details regarding data transmissions, cleaning, and algorithm processing within the administering electronic health records system are described in the Supplemental Appendix like this: "...Each week, the EHR routine saved all "valid" weight data and classified each person first by whether they had performed self-weighing on 0 days, 1 to 4 days, or 5 to 7 days (i.e., "daily"). The last valid weight in each week was used to calculate the weekly weight change, and each patient was classified as "making progress" (i.e., at least 0.5 pounds of weight loss in the past week) or not making progress. These data were stored in the patient's chart and used to update the "panel management" dashboard (above), as well as to trigger additional automated Mychart messages (below). To keep primary care clinicians aware of progress, the daily weight values for each patient were also stored as flowsheets, which were transmitted to the clinician's EHR "inbox," following existing workflows for routing lab results and communications among clinicians."

5-v) Ensure replicability by publishing the source code, and/or providing screenshots/screen-capture video, and/or providing flowcharts of the algorithms used

Ensure replicability by publishing the source code, and/or providing screenshots/screen-capture video, and/or providing flowcharts of the algorithms used. Replicability (i.e., other researchers should in principle be able to replicate the study) is a hallmark of scientific reporting.

	1	2	3	4	5	
subitem not at all important	◉	○	○	○	○	essential

Clear selection

Does your paper address subitem 5-v?

Copy and paste relevant sections from the manuscript (include quotes in quotation marks "like this" to indicate direct quotes from your manuscript), or elaborate on this item by providing additional information not in the ms, or briefly explain why the item is not applicable/relevant for your study

This is a preliminary study designed to demonstrate feasibility. Detailed intervention specifications will be compiled and made available once completed and studied in a more definitive trial design.

5-vi) Digital preservation

Digital preservation: Provide the URL of the application, but as the intervention is likely to change or disappear over the course of the years; also make sure the intervention is archived (Internet Archive, webcitation.org, and/or publishing the source code or screenshots/videos alongside the article). As pages behind login screens cannot be archived, consider creating demo pages which are accessible without login.

	1	2	3	4	5	
subitem not at all important	(selected)	○	○	○	○	essential

Clear selection

Does your paper address subitem 5-vi?

Copy and paste relevant sections from the manuscript (include quotes in quotation marks "like this" to indicate direct quotes from your manuscript), or elaborate on this item by providing additional information not in the ms, or briefly explain why the item is not applicable/relevant for your study

This is a preliminary study designed to demonstrate feasibility. Detailed intervention specifications will be compiled and made available once completed and studied in a more definitive trial design.

5-vii) Access

Access: Describe how participants accessed the application, in what setting/context, if they had to pay (or were paid) or not, whether they had to be a member of specific group. If known, describe how participants obtained “access to the platform and Internet” [1]. To ensure access for editors/reviewers/readers, consider to provide a “backdoor” login account or demo mode for reviewers/readers to explore the application (also important for archiving purposes, see vi).

	1	2	3	4	5	
subitem not at all important	○	○	●	○	○	essential

Clear selection

Does your paper address subitem 5-vii? *

Copy and paste relevant sections from the manuscript (include quotes in quotation marks "like this" to indicate direct quotes from your manuscript), or elaborate on this item by providing additional information not in the ms, or briefly explain why the item is not applicable/relevant for your study

All intervention components offered by the trial were free of charge to the patient participants. This is described like this: "...All 80 patients received, free-of-charge, a bundle of Basic Lifestyle Support (BLS) services..."

5-viii) Mode of delivery, features/functionalities/components of the intervention and comparator, and the theoretical framework

Describe mode of delivery, features/functionalities/components of the intervention and comparator, and the theoretical framework [6] used to design them (instructional strategy [1], behaviour change techniques, persuasive features, etc., see e.g., [7, 8] for terminology). This includes an in-depth description of the content (including where it is coming from and who developed it) [1],” whether [and how] it is tailored to individual circumstances and allows users to track their progress and receive feedback” [6]. This also includes a description of communication delivery channels and – if computer-mediated communication is a component – whether communication was synchronous or asynchronous [6]. It also includes information on presentation strategies [1], including page design principles, average amount of text on pages, presence of hyperlinks to other resources, etc. [1].

	1	2	3	4	5	
subitem not at all important	○	○	○	◉	○	essential

Clear selection

Does your paper address subitem 5-viii? *

Copy and paste relevant sections from the manuscript (include quotes in quotation marks "like this" to indicate direct quotes from your manuscript), or elaborate on this item by providing additional information not in the ms, or briefly explain why the item is not applicable/relevant for your study

Modes of delivery for multiple intervention components are described in detail, like this: "...they were shipped a wireless weight scale (e-Scale), which had been programmed to transmit weight data back to their electronic chart using cellular networks, at not cost to the patient. Each patient received a follow-up MyChart message, with simple instructions for using the scale to weigh themselves daily, as well as a “coupon code” to enroll free-of-charge in longitudinal lifestyle coaching and support resources offered by a regional fitness organization partner. Patients could choose to access the referral services in 2 formats: face-to-face access to a dietician and fitness coach at 11 facility locations or fully remote access to the same professionals via video chat with a smartphone, tablet, or personal computer. The face-to-face option provided full health club facility access, and the remote delivery option provided a suite of healthy food preparation and physical activity tips, tracking tools, and videos. Each patient also received instructions for messaging a dedicated nurse-coach at the primary care practice if they had problems using the support services or if they wanted more personalized support for reaching dietary, physical activity, or weight loss goals."

5-ix) Describe use parameters

Describe use parameters (e.g., intended “doses” and optimal timing for use). Clarify what instructions or recommendations were given to the user, e.g., regarding timing, frequency, heaviness of use, if any, or was the intervention used ad libitum.

	1	2	3	4	5	
subitem not at all important	○	○	○	◉	○	essential

Clear selection

Does your paper address subitem 5-ix?

Copy and paste relevant sections from the manuscript (include quotes in quotation marks "like this" to indicate direct quotes from your manuscript), or elaborate on this item by providing additional information not in the ms, or briefly explain why the item is not applicable/relevant for your study

Described like this: "...each patient in the CLS arm was sent an automated weekly MyChart message with coaching tips customized to their use of the e-Scale in the prior week, whether they had made weight loss progress (defined as ≥0.5 pounds of weight loss from the first to last weight measure during the prior week), and whether they had enrolled in referral-based services offered by the fitness partner. A practice nurse used an EHR dashboard to monitor the entire CLS group and attempted to call patients to offer customized “step-up” telephone coaching if either: (1) they had not used their scale in the prior week; or (2) for 2 consecutive weeks they had not achieved ≥0.5 pounds of weight loss."

5-x) Clarify the level of human involvement

Clarify the level of human involvement (care providers or health professionals, also technical assistance) in the e-intervention or as co-intervention (detail number and expertise of professionals involved, if any, as well as “type of assistance offered, the timing and frequency of the support, how it is initiated, and the medium by which the assistance is delivered”. It may be necessary to distinguish between the level of human involvement required for the trial, and the level of human involvement required for a routine application outside of a RCT setting (discuss under item 21 – generalizability).

	1	2	3	4	5	
subitem not at all important	○	○	◉	○	○	essential

Clear selection

Does your paper address subitem 5-x?

Copy and paste relevant sections from the manuscript (include quotes in quotation marks "like this" to indicate direct quotes from your manuscript), or elaborate on this item by providing additional information not in the ms, or briefly explain why the item is not applicable/relevant for your study

Described like this: "...A practice nurse used an EHR dashboard to monitor the entire CLS group and attempted to call patients to offer customized “step-up” telephone coaching if either: (1) they had not used their scale in the prior week; or (2) for 2 consecutive weeks they had not achieved ≥0.5 pounds of weight loss."

5-xi) Report any prompts/reminders used

Report any prompts/reminders used: Clarify if there were prompts (letters, emails, phone calls, SMS) to use the application, what triggered them, frequency etc. It may be necessary to distinguish between the level of prompts/reminders required for the trial, and the level of prompts/reminders for a routine application outside of a RCT setting (discuss under item 21 – generalizability).

	1	2	3	4	5	
subitem not at all important	○	○	○	◉	○	essential

Clear selection

Does your paper address subitem 5-xi? *

Copy and paste relevant sections from the manuscript (include quotes in quotation marks "like this" to indicate direct quotes from your manuscript), or elaborate on this item by providing additional information not in the ms, or briefly explain why the item is not applicable/relevant for your study

Described like this: "...each patient in the CLS arm was sent an automated weekly MyChart message with coaching tips customized to their use of the e-Scale in the prior week, whether they had made weight loss progress (defined as ≥0.5 pounds of weight loss from the first to last weight measure during the prior week), and whether they had enrolled in referral-based services offered by the fitness partner..." and in the Appendix like this: "...Automated coaching messages were designed at a 4th grade reading level and aimed to provide a source of supportive accountability while encouraging two goal behaviors under one's immediate control: (1) daily self-weighing and (2) engagement in additional forms of expert coaching services to support healthful diet and activity changes."

5-xii) Describe any co-interventions (incl. training/support)

Describe any co-interventions (incl. training/support): Clearly state any interventions that are provided in addition to the targeted eHealth intervention, as ehealth intervention may not be designed as stand-alone intervention. This includes training sessions and support [1]. It may be necessary to distinguish between the level of training required for the trial, and the level of training for a routine application outside of a RCT setting (discuss under item 21 – generalizability.

	1	2	3	4	5	
subitem not at all important	○	○	○	◉	○	essential

Clear selection

Does your paper address subitem 5-xii? *

Copy and paste relevant sections from the manuscript (include quotes in quotation marks "like this" to indicate direct quotes from your manuscript), or elaborate on this item by providing additional information not in the ms, or briefly explain why the item is not applicable/relevant for your study

Again, all components are described in detail, like this: "...Each patient received a follow-up MyChart message, with simple instructions for using the scale to weigh themselves daily, as well as a “coupon code” to enroll free-of-charge in longitudinal lifestyle coaching and support resources offered by a regional fitness organization partner. Patients could choose to access the referral services in 2 formats: face-to-face access to a dietician and fitness coach at 11 facility locations or fully remote access to the same professionals via video chat with a smartphone, tablet, or personal computer. The face-to-face option provided full health club facility access, and the remote delivery option provided a suite of healthy food preparation and physical activity tips, tracking tools, and videos. Each patient also received instructions for messaging a dedicated nurse-coach at the primary care practice if they had problems using the support services or if they wanted more personalized support for reaching dietary, physical activity, or weight loss goals..." and this: "...A practice nurse used an EHR dashboard to monitor the entire CLS group and attempted to call patients to offer customized “step-up” telephone coaching if either: (1) they had not used their scale in the prior week; or (2) for 2 consecutive weeks they had not achieved ≥0.5 pounds of weight loss."

6a) Completely defined pre-specified primary and secondary outcome measures, including how and when they were assessed

Does your paper address CONSORT subitem 6a? *

Copy and paste relevant sections from the manuscript (include quotes in quotation marks "like this" to indicate direct quotes from your manuscript), or elaborate on this item by providing additional information not in the ms, or briefly explain why the item is not applicable/relevant for your study

Like this: "The primary quantitative outcome was the percentage of study patients achieving weight loss of ≥5% of their baseline body weight at 6 ±3 months. Additional outcomes included: the feasibility of automated trial eligibility determination, enrollment, and randomization; feasibility of pragmatic weight data capture from office visits and electronic scales; utilization of different intervention components; health system costs to implement each intervention component; patient perceptions regarding the appropriateness, ease of access, ease of use, acceptability, desirability, credibility, and usefulness of the intervention components."

6a-i) Online questionnaires: describe if they were validated for online use and apply CHERRIES items to describe how the questionnaires were designed/deployed

If outcomes were obtained through online questionnaires, describe if they were validated for online use and apply CHERRIES items to describe how the questionnaires were designed/deployed [9].

	1	2	3	4	5	
subitem not at all important	◉	○	○	○	○	essential

Clear selection

Does your paper address subitem 6a-i?

Copy and paste relevant sections from manuscript text

Not applicable. We did not use online questionnaires.

6a-ii) Describe whether and how “use” (including intensity of use/dosage) was defined/measured/monitored

Describe whether and how “use” (including intensity of use/dosage) was defined/measured/monitored (logins, logfile analysis, etc.). Use/adoption metrics are important process outcomes that should be reported in any ehealth trial.

	1	2	3	4	5	
subitem not at all important	○	○	○	◉	○	essential

Clear selection

Does your paper address subitem 6a-ii?

Copy and paste relevant sections from manuscript text

Like this: "...Multiple data sources were used to describe utilization of the different intervention components. Input from health system leaders and study team members identified the specific personnel, supplies, contract services, and facility and administrative inputs needed to implement each intervention component. Electronic scales provided data on the frequency of weight self-monitoring. Administrative data systems captured the number and type of contacts made between nurses and study participants. The partnering fitness network also provided data about patient participation in the two coaching support options and facility use."

6a-iii) Describe whether, how, and when qualitative feedback from participants was obtained

Describe whether, how, and when qualitative feedback from participants was obtained (e.g., through emails, feedback forms, interviews, focus groups).

	1	2	3	4	5	
subitem not at all important	○	○	○	○	◉	essential

Clear selection

Does your paper address subitem 6a-iii?

Copy and paste relevant sections from manuscript text

Like this: "...Patient reported data were collected by semi-structured telephone interviews with 15 randomly-selected patients who were recruited beginning approximately 12 weeks after their enrollment in the feasibility trial (see recruitment above). Interviews assessed perceptions regarding the appropriateness, ease of access, ease of use, acceptability, desirability, credibility, and usefulness of the intervention components in helping the patient achieve health related goals."

6b) Any changes to trial outcomes after the trial commenced, with reasons

Does your paper address CONSORT subitem 6b? *

Copy and paste relevant sections from the manuscript (include quotes in quotation marks "like this" to indicate direct quotes from your manuscript), or elaborate on this item by providing additional information not in the ms, or briefly explain why the item is not applicable/relevant for your study

Not applicable. no changes were made after the trial commenced.

7a) How sample size was determined

NPT: When applicable, details of whether and how the clustering by care provides or centers was addressed

7a-i) Describe whether and how expected attrition was taken into account when calculating the sample size

Describe whether and how expected attrition was taken into account when calculating the sample size.

	1	2	3	4	5	
subitem not at all important	◉	○	○	○	○	essential

Clear selection

Does your paper address subitem 7a-i?

Copy and paste relevant sections from manuscript title (include quotes in quotation marks "like this" to indicate direct quotes from your manuscript), or elaborate on this item by providing additional information not in the ms, or briefly explain why the item is not applicable/relevant for your study

This manuscript reports results of a small feasibility trial. It was not designed through power analysis to enroll subjects sufficient to test a specific hypothesized outcome difference. It is described like this: "...until the target sample size of 80 patients had affirmed interest in receiving the intervention services. This sample size was believed sufficient to demonstrate feasibility and fideltity of pragmatic randomization and data collection procedures, to test the deployment of all variations of the patient messages, and to collect robust qualitative data about intervention perceptions."

7b) When applicable, explanation of any interim analyses and stopping guidelines

Does your paper address CONSORT subitem 7b? *

Copy and paste relevant sections from the manuscript (include quotes in quotation marks "like this" to indicate direct quotes from your manuscript), or elaborate on this item by providing additional information not in the ms, or briefly explain why the item is not applicable/relevant for your study

This is not at all applicable to this small feasibility trial design.

8a) Method used to generate the random allocation sequence
NPT: When applicable, how care providers were allocated to each trial group

Does your paper address CONSORT subitem 8a? *

Copy and paste relevant sections from the manuscript (include quotes in quotation marks "like this" to indicate direct quotes from your manuscript), or elaborate on this item by providing additional information not in the ms, or briefly explain why the item is not applicable/relevant for your study

Random allocation was based on a permutation of each individual patient's unique health system patient ID. The electronic health record was programmed to assign a different intervention group assignment based on different possible numbering sequences. The group assignment then initiated a series of health record routines that involved deploying a different sequences of outbound messages that contained different information for each group. Given the small size of the feasibility trial and that it wasn't designed to evaluate between group differences in any outcomes, the full details for the randomization procedure are not described in this report but will be in subsequent, larger-scale trials that rely on random group assignment for the reported study outcomes.

8b) Type of randomisation; details of any restriction (such as blocking and block size)

Does your paper address CONSORT subitem 8b? *

Copy and paste relevant sections from the manuscript (include quotes in quotation marks "like this" to indicate direct quotes from your manuscript), or elaborate on this item by providing additional information not in the ms, or briefly explain why the item is not applicable/relevant for your study

The study used simple randomization. See above. These details are beyond the scope of the current feasibility trial report.

9) Mechanism used to implement the random allocation sequence (such as sequentially numbered containers), describing any steps taken to conceal the sequence until interventions were assigned

Does your paper address CONSORT subitem 9? *

Copy and paste relevant sections from the manuscript (include quotes in quotation marks "like this" to indicate direct quotes from your manuscript), or elaborate on this item by providing additional information not in the ms, or briefly explain why the item is not applicable/relevant for your study

Again. N/A. Random allocation was based on a permutation of each individual patient's unique health system patient ID. The electronic health record was programmed to assign a different intervention group assignment based on different possible numbering sequences. The group assignment then initiated a series of health record routines that involved deploying a different sequences of outbound messages that contained different information for each group. Given the small size of the feasibility trial and that it wasn't designed to evaluate between group differences in any outcomes, the full details for the randomization procedure are not described in this report but will be in subsequent, larger-scale trials that rely on random group assignment for the reported study outcomes.

10) Who generated the random allocation sequence, who enrolled participants, and who assigned participants to interventions

Does your paper address CONSORT subitem 10? *

Copy and paste relevant sections from the manuscript (include quotes in quotation marks "like this" to indicate direct quotes from your manuscript), or elaborate on this item by providing additional information not in the ms, or briefly explain why the item is not applicable/relevant for your study

Random allocation was based on a permutation of each individual patient's unique health system patient ID. The electronic health record was programmed to assign a different intervention group assignment based on different possible numbering sequences. The group assignment then initiated a series of health record routines that involved deploying a different sequences of outbound messages that contained different information for each group. Given the small size of the feasibility trial and that it wasn't designed to evaluate between group differences in any outcomes, the full details for the randomization procedure are not described in this report but will be in subsequent, larger-scale trials that rely on random group assignment for the reported study outcomes.

11a) If done, who was blinded after assignment to interventions (for example, participants, care providers, those assessing outcomes) and how

NPT: Whether or not administering co-interventions were blinded to group assignment

11a-i) Specify who was blinded, and who wasn't

Specify who was blinded, and who wasn't. Usually, in web-based trials it is not possible to blind the participants [1, 3] (this should be clearly acknowledged), but it may be possible to blind outcome assessors, those doing data analysis or those administering co-interventions (if any).

	1	2	3	4	5	
subitem not at all important	(●)	( )	( )	( )	( )	essential

Clear selection

Does your paper address subitem 11a-i? *

Copy and paste relevant sections from the manuscript (include quotes in quotation marks "like this" to indicate direct quotes from your manuscript), or elaborate on this item by providing additional information not in the ms, or briefly explain why the item is not applicable/relevant for your study

This study focuses on feasibility outcomes that do not rely on blinded intervention receipt or delivery. Because all intervention components are service delivery interventions, they are all inherently "unblinded" to participants. In other words, participants know who they are interacting with, what messages they are reading, and what IT they are interacting with. However, it is important that we use a design where study subjects are enrolled based on their interest in receiving and giving feedback about novel health promotion resources and services that the health system is trying to develop and improve. Because they are not told there will be random assignment to different intervention conditions, there should be no differences in outcome expectations based on group assignment. Both study arms are "active" and patients know what they receive but not that other patients may receive different interventions.

11a-ii) Discuss e.g., whether participants knew which intervention was the “intervention of interest” and which one was the “comparator”

Informed consent procedures (4a-ii) can create biases and certain expectations - discuss e.g., whether participants knew which intervention was the “intervention of interest” and which one was the “comparator”.

	1	2	3	4	5	
subitem not at all important	○	○	○	◉	○	essential

Clear selection

Does your paper address subitem 11a-ii?

Copy and paste relevant sections from the manuscript (include quotes in quotation marks "like this" to indicate direct quotes from your manuscript), or elaborate on this item by providing additional information not in the ms, or briefly explain why the item is not applicable/relevant for your study

All study subjects are enrolled based on their interest in receiving and giving feedback about novel health promotion resources and services that the health system is trying to develop and improve. Because they are not told there will be random assignment to different intervention conditions, there should be no differences in outcome expectations based on group assignment. Both study arms are "active" and patients know what they receive but not that other patients may receive different interventions.

11b) If relevant, description of the similarity of interventions

(this item is usually not relevant for ehealth trials as it refers to similarity of a placebo or sham intervention to a active medication/intervention)

Does your paper address CONSORT subitem 11b? *

Copy and paste relevant sections from the manuscript (include quotes in quotation marks "like this" to indicate direct quotes from your manuscript), or elaborate on this item by providing additional information not in the ms, or briefly explain why the item is not applicable/relevant for your study

Both treatment conditions involve receipt of a personal electronic scale, recurring messages received through an electronic health record patient portal, and access to practice nurses for more individualized coaching. The two groups in this regard have similar attention. There is no arm involving no intervention or know attention. This is described in detail.

12a) Statistical methods used to compare groups for primary and secondary outcomes

NPT: When applicable, details of whether and how the clustering by care providers or centers was addressed

Does your paper address CONSORT subitem 12a? *

Copy and paste relevant sections from the manuscript (include quotes in quotation marks "like this" to indicate direct quotes from your manuscript), or elaborate on this item by providing additional information not in the ms, or briefly explain why the item is not applicable/relevant for your study

Described like this: "....We used chi-square tests to compare all patients randomized to CLS and BLS arms on the proportion that reached the dichotomous outcomes of any weight loss at 6 months and the intervention goal threshold of ≥5% weight loss at 6 months."

12a-i) Imputation techniques to deal with attrition / missing values

Imputation techniques to deal with attrition / missing values: Not all participants will use the intervention/comparator as intended and attrition is typically high in ehealth trials. Specify how participants who did not use the application or dropped out from the trial were treated in the statistical analysis (a complete case analysis is strongly discouraged, and simple imputation techniques such as LOCF may also be problematic [4]).

	1	2	3	4	5	
subitem not at all important	○	◉	○	○	○	essential

Clear selection

Does your paper address subitem 12a-i? *

Copy and paste relevant sections from the manuscript (include quotes in quotation marks "like this" to indicate direct quotes from your manuscript), or elaborate on this item by providing additional information not in the ms, or briefly explain why the item is not applicable/relevant for your study

Our study evaluated preliminary weight change outcomes within the small feasibility sample. As a result of studying the feasibility of weight data capture from the electronic health record, we learned that, after considering all data sources, a valid weight reading was missing at 6 months for 3 of 80 patients. We reported it like this: "...our pragmatic data capture strategy enabled estimation of a 6-month weight change for 77 (96%) of all trial participants." Our weight outcome of interest was the probability of achieving 5% or greater weight loss at 6 months. Rather than to impute the 3 missing weight values (for which there is much disagreement about best practices), we conservatively assumed that all 3 of those people failed to reach the 5% weight loss threshold. This is technically not imputation.

12b) Methods for additional analyses, such as subgroup analyses and adjusted analyses

Does your paper address CONSORT subitem 12b? *

Copy and paste relevant sections from the manuscript (include quotes in quotation marks "like this" to indicate direct quotes from your manuscript), or elaborate on this item by providing additional information not in the ms, or briefly explain why the item is not applicable/relevant for your study

This was a small feasibility trial. We did not include subgroup analyses.

X26) REB/IRB Approval and Ethical Considerations [recommended as subheading under "Methods"] (not a CONSORT item)

X26-i) Comment on ethics committee approval

	1	2	3	4	5	
subitem not at all important	○	○	○	●	○	essential

Clear selection

Does your paper address subitem X26-i?

Copy and paste relevant sections from the manuscript (include quotes in quotation marks "like this" to indicate direct quotes from your manuscript), or elaborate on this item by providing additional information not in the ms, or briefly explain why the item is not applicable/relevant for your study

reported like this: "All study procedures were performed in accordance with the Declaration of Helsinki. Study procedures, including pragmatic enrollment and randomization under a waiver of written informed consent for research, were approved by the Northwestern University IRB (STU00207153); the pilot trial was registered and posted on 25/06/2019 at ClinicalTrials.gov (NCT03998046)."

x26-ii) Outline informed consent procedures

Outline informed consent procedures e.g., if consent was obtained offline or online (how? Checkbox, etc.?), and what information was provided (see 4a-ii). See [6] for some items to be included in informed consent documents.

	1	2	3	4	5	
subitem not at all important	(●)	○	○	○	○	essential

Clear selection

Does your paper address subitem X26-ii?

Copy and paste relevant sections from the manuscript (include quotes in quotation marks "like this" to indicate direct quotes from your manuscript), or elaborate on this item by providing additional information not in the ms, or briefly explain why the item is not applicable/relevant for your study

Like this: "...Study procedures, including pragmatic enrollment and randomization under a waiver of written informed consent for research, were approved by the Northwestern University IRB (STU00207153)"

X26-iii) Safety and security procedures

Safety and security procedures, incl. privacy considerations, and any steps taken to reduce the likelihood or detection of harm (e.g., education and training, availability of a hotline)

	1	2	3	4	5	
subitem not at all important	(●)	○	○	○	○	essential

Clear selection

Does your paper address subitem X26-iii?

Copy and paste relevant sections from the manuscript (include quotes in quotation marks "like this" to indicate direct quotes from your manuscript), or elaborate on this item by providing additional information not in the ms, or briefly explain why the item is not applicable/relevant for your study

Our IRB requires that all members of the research team complete human subjects' research training and that the certification is updated regularly. the study was a "minimal risk" protocol with a data safety and monitoring plan but no specific safety endpoints.

RESULTS

13a) For each group, the numbers of participants who were randomly assigned, received intended treatment, and were analysed for the primary outcome

NPT: The number of care providers or centers performing the intervention in each group and the number of patients treated by each care provider in each center

Does your paper address CONSORT subitem 13a? *

Copy and paste relevant sections from the manuscript (include quotes in quotation marks "like this" to indicate direct quotes from your manuscript), or elaborate on this item by providing additional information not in the ms, or briefly explain why the item is not applicable/relevant for your study

Sample accrual is described like this: "...and 80 (18.8%) affirmed interest in the weight loss goal (Figure 1)." Figure 1 is a Consort flow figure that clearly depicts numbers randomized and analyzed.

13b) For each group, losses and exclusions after randomisation, together with reasons

Does your paper address CONSORT subitem 13b? (NOTE: Preferably, this is shown in a CONSORT flow diagram) *

Copy and paste relevant sections from the manuscript (include quotes in quotation marks "like this" to indicate direct quotes from your manuscript), or elaborate on this item by providing additional information not in the ms, or briefly explain why the item is not applicable/relevant for your study

There were no losses or exclusions after randomization.

13b-i) Attrition diagram

Strongly recommended: An attrition diagram (e.g., proportion of participants still logging in or using the intervention/comparator in each group plotted over time, similar to a survival curve) or other figures or tables demonstrating usage/dose/engagement.

	1	2	3	4	5	
subitem not at all important	○	◉	○	○	○	essential

Clear selection

Does your paper address subitem 13b-i?

Copy and paste relevant sections from the manuscript or cite the figure number if applicable (include quotes in quotation marks "like this" to indicate direct quotes from your manuscript), or elaborate on this item by providing additional information not in the ms, or briefly explain why the item is not applicable/relevant for your study

Our study follows an intent to treat principle. Since no person needed to volunteer to enroll, no person disenrolled. Attrition, as defined by research follow-up was not an issue in this trial. Figure 1 demonstrates this, as does the text. Dose of engagement in different intervention components is a separate issue, and it is described in its own result section, like this: "Over the 12-week MyChart messaging phase of the intervention, 11 (14%) study patients did not use their scales for self-weighing, 7 (9%) participated in weighing in some but not all weeks, and 62 (78%) engaged in self-weighing during all 12 weeks. Self-weighing daily (5 to 7 days per week) was performed by 26 (33%) patients during all 12 weeks; more patients assigned to CLS (customized weekly messaging) performed daily self-weighing in all weeks (18 of 42 patients (43%) versus 8 of 38 (21%) patients offered only BLS, P=.06). Overall, 36 (45%) patients (14 (37%) in the BLS arm and 22 (52%) in the CLS arm (P=.18) enrolled in one of the two referral-based coaching platforms offered by the fitness industry partner; 22 (28%) elected the face-to-face option and 14 (18%) elected the fully remote delivery option. Patients enrolled in these services between 15 and 77 days after receiving the initial outreach MyChart message. Access to fitness facilities (i.e., the face-to-face delivery option) was disrupted by a coronavirus pandemic stay-at-home order beginning the third week of March 2020. At that time, patients had been enrolled in the referral-based services for 19 to 75 days; the 22 patients in face-to-face support had completed an average of 3 (range 1 to 6) individual coaching sessions and attended a partnering fitness facility 0 to 24 times; the 14 patients receiving fully remote support had completed an average of 4 (range 2 to 5) individual coaching sessions prior to the pandemic but were able to complete an average of 3 additional (range 0 to 5) coaching sessions after the pandemic began. Among patients randomized to CLS, 41 (98%) received at least one step-up telephonic nurse coaching call; 19 (45%) received ≥5 nurse calls (maximum 11 calls received) over the 12-week intensive support phase."

14a) Dates defining the periods of recruitment and follow-up

Does your paper address CONSORT subitem 14a? *

Copy and paste relevant sections from the manuscript (include quotes in quotation marks "like this" to indicate direct quotes from your manuscript), or elaborate on this item by providing additional information not in the ms, or briefly explain why the item is not applicable/relevant for your study

Described like this: "...Eligible patients were enrolled from December 2019 – January 2020; data were collected through January 2021. The experimental services were offered to patients beginning January 2020, and access to some services (details below) were interrupted by the coronavirus pandemic stay-at-home order in March 2020."

14a-i) Indicate if critical “secular events” fell into the study period

Indicate if critical “secular events” fell into the study period, e.g., significant changes in Internet resources available or “changes in computer hardware or Internet delivery resources”

	1	2	3	4	5	
subitem not at all important	○	○	◉	○	○	essential

Clear selection

Does your paper address subitem 14a-i?

Copy and paste relevant sections from the manuscript (include quotes in quotation marks "like this" to indicate direct quotes from your manuscript), or elaborate on this item by providing additional information not in the ms, or briefly explain why the item is not applicable/relevant for your study

Described like this: "...Access to fitness facilities (i.e., the face-to-face delivery option) was disrupted by a coronavirus pandemic stay-at-home order beginning the third week of March 2020. At that time, patients had been enrolled in the referral-based services for 19 to 75 days; the 22 patients in face-to-face support had completed an average of 3 (range 1 to 6) individual coaching sessions and attended a partnering fitness facility 0 to 24 times; the 14 patients receiving fully remote support had completed an average of 4 (range 2 to 5) individual coaching sessions prior to the pandemic but were able to complete an average of 3 additional (range 0 to 5) coaching sessions after the pandemic began."

14b) Why the trial ended or was stopped (early)

Does your paper address CONSORT subitem 14b? *

Copy and paste relevant sections from the manuscript (include quotes in quotation marks "like this" to indicate direct quotes from your manuscript), or elaborate on this item by providing additional information not in the ms, or briefly explain why the item is not applicable/relevant for your study

The trial was not stopped early. It was completed as planned, with some disruption of intervention components by pandemic, described like this: "...The experimental services were offered to patients beginning January 2020, and access to some services (details below) were interrupted by the coronavirus pandemic stay-at-home order in March 2020."

15) A table showing baseline demographic and clinical characteristics for each group

NPT: When applicable, a description of care providers (case volume, qualification, expertise, etc.) and centers (volume) in each group

Does your paper address CONSORT subitem 15? *

Copy and paste relevant sections from the manuscript (include quotes in quotation marks "like this" to indicate direct quotes from your manuscript), or elaborate on this item by providing additional information not in the ms, or briefly explain why the item is not applicable/relevant for your study

This is all detailed clearly in Table 1.

15-i) Report demographics associated with digital divide issues

In ehealth trials it is particularly important to report demographics associated with digital divide issues, such as age, education, gender, social-economic status, computer/Internet/ehealth literacy of the participants, if known.

	1	2	3	4	5	
subitem not at all important	○	○	◉	○	○	essential

Clear selection

Does your paper address subitem 15-i? *

Copy and paste relevant sections from the manuscript (include quotes in quotation marks "like this" to indicate direct quotes from your manuscript), or elaborate on this item by providing additional information not in the ms, or briefly explain why the item is not applicable/relevant for your study

We report available data on sex, age, and race/ethnicity for all 80 study patients. This is included in Table 1. All patients approached were enrolled in the health system's patient portal for remote communications and results access. At the time of the study, about 80% of all patients in this practice were enrolled. There are modest differences in patient portal enrollment by age, sex, and race. Patients more likely to remain not enrolled in this "MyChart" portal identify as non-Hispanic Black and older. Table 1 of our report shows that men enrolled in the patient portal were less likely than women to engage in the support services, as were adults over age 65 and those identifying as white race. The study was not designed to determine if this engagement was due to interest or literacy regarding technology or because of interest in receiving support for a weight loss goal. Our data sources do not include information for income or education level.

16) For each group, number of participants (denominator) included in each analysis and whether the analysis was by original assigned groups

16-i) Report multiple “denominators” and provide definitions

Report multiple “denominators” and provide definitions: Report N’s (and effect sizes) “across a range of study participation [and use] thresholds” [1], e.g., N exposed, N consented, N used more than x times, N used more than y weeks, N participants “used” the intervention/comparator at specific pre-defined time points of interest (in absolute and relative numbers per group). Always clearly define “use” of the intervention.

	1	2	3	4	5	
subitem not at all important	(●)	( )	( )	( )	( )	essential

Clear selection

Does your paper address subitem 16-i? *

Copy and paste relevant sections from the manuscript (include quotes in quotation marks "like this" to indicate direct quotes from your manuscript), or elaborate on this item by providing additional information not in the ms, or briefly explain why the item is not applicable/relevant for your study

This is a small feasibility trial that was not designed to make statistical comparisons in any study outcome between treatment arms. Randomization was only included to demonstrate feasibility of the imbedded randomization procedure within an electronic health record system.

16-ii) Primary analysis should be intent-to-treat

Primary analysis should be intent-to-treat, secondary analyses could include comparing only “users”, with the appropriate caveats that this is no longer a randomized sample (see 18-i).

	1	2	3	4	5	
subitem not at all important	( )	( )	( )	( )	(●)	essential

Clear selection

Does your paper address subitem 16-ii?

Copy and paste relevant sections from the manuscript (include quotes in quotation marks "like this" to indicate direct quotes from your manuscript), or elaborate on this item by providing additional information not in the ms, or briefly explain why the item is not applicable/relevant for your study

Like this: "...Overall, 48 (60%) of 80 participants had lower weight at follow up than at baseline; 12 (15%) exhibited a weight loss of ≥5% of baseline weight."

17a) For each primary and secondary outcome, results for each group, and the estimated effect size and its precision (such as 95% confidence interval)

Does your paper address CONSORT subitem 17a? *

Copy and paste relevant sections from the manuscript (include quotes in quotation marks "like this" to indicate direct quotes from your manuscript), or elaborate on this item by providing additional information not in the ms, or briefly explain why the item is not applicable/relevant for your study

The results of this feasibility study are descriptive and not powered for analysis of between group effect sizes in any continuous outcome.

17a-i) Presentation of process outcomes such as metrics of use and intensity of use

In addition to primary/secondary (clinical) outcomes, the presentation of process outcomes such as metrics of use and intensity of use (dose, exposure) and their operational definitions is critical. This does not only refer to metrics of attrition (13-b) (often a binary variable), but also to more continuous exposure metrics such as “average session length”. These must be accompanied by a technical description how a metric like a “session” is defined (e.g., timeout after idle time) [1] (report under item 6a).

	1	2	3	4	5	
subitem not at all important	○	○	○	◉	○	essential

Clear selection

Does your paper address subitem 17a-i?

Copy and paste relevant sections from the manuscript (include quotes in quotation marks "like this" to indicate direct quotes from your manuscript), or elaborate on this item by providing additional information not in the ms, or briefly explain why the item is not applicable/relevant for your study

Like this: "Overall, 36 (45%) patients (14 (37%) in the BLS arm and 22 (52%) in the CLS arm (P=.18) enrolled in one of the two referral-based coaching platforms offered by the fitness industry partner; 22 (28%) elected the face-to-face option and 14 (18%) elected the fully remote delivery option. Patients enrolled in these services between 15 and 77 days after receiving the initial outreach MyChart message. Access to fitness facilities (i.e., the face-to-face delivery option) was disrupted by a coronavirus pandemic stay-at-home order beginning the third week of March 2020. At that time, patients had been enrolled in the referral-based services for 19 to 75 days; the 22 patients in face-to-face support had completed an average of 3 (range 1 to 6) individual coaching sessions and attended a partnering fitness facility 0 to 24 times; the 14 patients receiving fully remote support had completed an average of 4 (range 2 to 5) individual coaching sessions prior to the pandemic but were able to complete an average of 3 additional (range 0 to 5) coaching sessions after the pandemic began. Among patients randomized to CLS, 41 (98%) received at least one step-up telephonic nurse coaching call; 19 (45%) received ≥5 nurse calls (maximum 11 calls received) over the 12-week intensive support phase."

17b) For binary outcomes, presentation of both absolute and relative effect sizes is recommended

Does your paper address CONSORT subitem 17b? *

Copy and paste relevant sections from the manuscript (include quotes in quotation marks "like this" to indicate direct quotes from your manuscript), or elaborate on this item by providing additional information not in the ms, or briefly explain why the item is not applicable/relevant for your study

The study is not designed to make between-group comparisons of absolute or relative effects.

18) Results of any other analyses performed, including subgroup analyses and adjusted analyses, distinguishing pre-specified from exploratory

Does your paper address CONSORT subitem 18? *

Copy and paste relevant sections from the manuscript (include quotes in quotation marks "like this" to indicate direct quotes from your manuscript), or elaborate on this item by providing additional information not in the ms, or briefly explain why the item is not applicable/relevant for your study

This is not applicable to our study.

18-i) Subgroup analysis of comparing only users

A subgroup analysis of comparing only users is not uncommon in ehealth trials, but if done, it must be stressed that this is a self-selected sample and no longer an unbiased sample from a randomized trial (see 16-iii).

	1	2	3	4	5	
subitem not at all important	(●)	○	○	○	○	essential

Clear selection

Does your paper address subitem 18-i?

Copy and paste relevant sections from the manuscript (include quotes in quotation marks "like this" to indicate direct quotes from your manuscript), or elaborate on this item by providing additional information not in the ms, or briefly explain why the item is not applicable/relevant for your study

We did not perform subgroup analysis of only users

19) All important harms or unintended effects in each group

(for specific guidance see CONSORT for harms)

Does your paper address CONSORT subitem 19? *

Copy and paste relevant sections from the manuscript (include quotes in quotation marks "like this" to indicate direct quotes from your manuscript), or elaborate on this item by providing additional information not in the ms, or briefly explain why the item is not applicable/relevant for your study

The study did not have anticipated harms. It did not identify any unanticipated harms.

19-i) Include privacy breaches, technical problems

Include privacy breaches, technical problems. This does not only include physical "harm" to participants, but also incidents such as perceived or real privacy breaches [1], technical problems, and other unexpected/unintended incidents. "Unintended effects" also includes unintended positive effects [2].

	1	2	3	4	5	
subitem not at all important	○	◉	○	○	○	essential

Clear selection

Does your paper address subitem 19-i?

Copy and paste relevant sections from the manuscript (include quotes in quotation marks "like this" to indicate direct quotes from your manuscript), or elaborate on this item by providing additional information not in the ms, or briefly explain why the item is not applicable/relevant for your study

The IT components of the intervention are all imbedded in the electronic health record of the partnering health system. No privacy breach was reported during the study. The study includes description of a lengthy design, prototyping, and refinement phase prior to the trial. Several technical challenges were encountered and overcome before the trial was launched. no technical challenges were identified during the trial period.

19-ii) Include qualitative feedback from participants or observations from staff/researchers

Include qualitative feedback from participants or observations from staff/researchers, if available, on strengths and shortcomings of the application, especially if they point to unintended/unexpected effects or uses. This includes (if available) reasons for why people did or did not use the application as intended by the developers.

	1	2	3	4	5	
subitem not at all important	○	○	○	◉	○	essential

Clear selection

Does your paper address subitem 19-ii?

Copy and paste relevant sections from the manuscript (include quotes in quotation marks "like this" to indicate direct quotes from your manuscript), or elaborate on this item by providing additional information not in the ms, or briefly explain why the item is not applicable/relevant for your study

Like this: "...Patient perceptions are summarized below; representative quotes and considerations for design refinements are organized in Additional File Table 2.
Most patients described the Mychart messages and website resources as generally easy to understand and navigate. However, some reported feeling frustrated or less engaged by not having a clear understanding upfront for how the different intervention components were coordinated or should be utilized. Trust in the person or organization providing lifestyle support services emerged as an important theme."

DISCUSSION

22) Interpretation consistent with results, balancing benefits and harms, and considering other relevant evidence

NPT: In addition, take into account the choice of the comparator, lack of or partial blinding, and unequal expertise of care providers or centers in each group

22-i) Restate study questions and summarize the answers suggested by the data, starting with primary outcomes and process outcomes (use)

Restate study questions and summarize the answers suggested by the data, starting with primary outcomes and process outcomes (use).

	1	2	3	4	5	
subitem not at all important	○	○	○	◉	○	essential

Clear selection

Does your paper address subitem 22-i? *

Copy and paste relevant sections from the manuscript (include quotes in quotation marks "like this" to indicate direct quotes from your manuscript), or elaborate on this item by providing additional information not in the ms, or briefly explain why the item is not applicable/relevant for your study

described like this: "...This preliminary study was conducted to demonstrate the feasibility of several pragmatic practice components and implementation strategies for primary care offices to support the core components of intensive lifestyle interventions. We also sought to demonstrate the feasibility of capturing and analyzing weight data from two secondary sources, the electronic health record and e-scales, as an important biometric outcome for future research..." and this: "...Among those 80 patients, 24 (30%) achieved ≥5% weight loss in the first 12 weeks and, despite the disruptive impacts of a pandemic, 12 (15%) still exhibited ≥5% weight loss at 6 months..." and this: "...Though this small feasibility trial did not demonstrate whether patients are more likely to reach weight loss goals when provided these more customized services, this approach warrants further study."

22-ii) Highlight unanswered new questions, suggest future research

Highlight unanswered new questions, suggest future research.

	1	2	3	4	5	
subitem not at all important	○	○	○	○	◉	essential

Clear selection

Does your paper address subitem 22-ii?

Copy and paste relevant sections from the manuscript (include quotes in quotation marks "like this" to indicate direct quotes from your manuscript), or elaborate on this item by providing additional information not in the ms, or briefly explain why the item is not applicable/relevant for your study

Like this: "...This preliminary research indicates that strategies to implement ILI core components are feasible for primary care practices and generally acceptable to patients. Further research should seek to optimize implementation strategies to maximize feasibility, acceptability, sustainability, and reach, as well as to demonstrate effectiveness and cost-effectiveness compared with alternative service models."

20) Trial limitations, addressing sources of potential bias, imprecision, and, if relevant, multiplicity of analyses

20-i) Typical limitations in ehealth trials

Typical limitations in ehealth trials: Participants in ehealth trials are rarely blinded. Ehealth trials often look at a multiplicity of outcomes, increasing risk for a Type I error. Discuss biases due to non-use of the intervention/usability issues, biases through informed consent procedures, unexpected events.

	1	2	3	4	5	
subitem not at all important	(●)	( )	( )	( )	( )	essential

Clear selection

Does your paper address subitem 20-i? *

Copy and paste relevant sections from the manuscript (include quotes in quotation marks "like this" to indicate direct quotes from your manuscript), or elaborate on this item by providing additional information not in the ms, or briefly explain why the item is not applicable/relevant for your study

Internal validity limitations were really not relevant in this small, preliminary research study.

21) Generalisability (external validity, applicability) of the trial findings

NPT: External validity of the trial findings according to the intervention, comparators, patients, and care providers or centers involved in the trial

21-i) Generalizability to other populations

Generalizability to other populations: In particular, discuss generalizability to a general Internet population, outside of a RCT setting, and general patient population, including applicability of the study results for other organizations

	1	2	3	4	5	
subitem not at all important	○	○	●	○	○	essential

Clear selection

Does your paper address subitem 21-i?

Copy and paste relevant sections from the manuscript (include quotes in quotation marks "like this" to indicate direct quotes from your manuscript), or elaborate on this item by providing additional information not in the ms, or briefly explain why the item is not applicable/relevant for your study

Like this: "...Possible disadvantages are that the individual components may not prove as effective in isolation, and additional strategies must be developed to coordinate staff roles, technologies, and patient transitions among the practice components. Some patients may still face access barriers if, for example, referral-based resources are limited or health payers do not provide reimbursement for remote weight monitoring or lifestyle counseling activities by non-physicians.[43, 44] Although our approach offers lower technology intervention options, such as nurse-delivered telephone counseling and facility-based resources offered by a partnering fitness organization, these interventions may still have different effects based on technology preferences and literacy. It will be important for future research to evaluate these dimensions of implementation in the context of a larger and more definitive effectiveness trial."

21-ii) Discuss if there were elements in the RCT that would be different in a routine application setting

Discuss if there were elements in the RCT that would be different in a routine application setting (e.g., prompts/reminders, more human involvement, training sessions or other co-interventions) and what impact the omission of these elements could have on use, adoption, or outcomes if the intervention is applied outside of a RCT setting.

	1	2	3	4	5	
subitem not at all important	○	○	◉	○	○	essential

Clear selection

Does your paper address subitem 21-ii?

Copy and paste relevant sections from the manuscript (include quotes in quotation marks "like this" to indicate direct quotes from your manuscript), or elaborate on this item by providing additional information not in the ms, or briefly explain why the item is not applicable/relevant for your study

Our study identified, enrolled, and randomized individuals "pragmatically." The study sample is therefore generalizable to anyone who would engage in the service components when offered during routine practice. There is no volunteer bias. All communications came from service professionals and used existing clinical messaging channels, so the engagement approaches and other implementation features will not change between our study and real world practice.

OTHER INFORMATION

23) Registration number and name of trial registry

Does your paper address CONSORT subitem 23? *

Copy and paste relevant sections from the manuscript (include quotes in quotation marks "like this" to indicate direct quotes from your manuscript), or elaborate on this item by providing additional information not in the ms, or briefly explain why the item is not applicable/relevant for your study

Like this: "...the pilot trial was registered and posted on 25/06/2019 at ClinicalTrials.gov (NCT03998046)."

24) Where the full trial protocol can be accessed, if available

Does your paper address CONSORT subitem 24? *

Cite a Multimedia Appendix, other reference, or copy and paste relevant sections from the manuscript (include quotes in quotation marks "like this" to indicate direct quotes from your manuscript), or elaborate on this item by providing additional information not in the ms, or briefly explain why the item is not applicable/relevant for your study

Other than the clinicaltrials.gov record, additional protocol details, manuals, and other intervention resources and documentation will be released after definitive testing commences in the future, full scale trial that follows this preliminary study.

25) Sources of funding and other support (such as supply of drugs), role of funders

Does your paper address CONSORT subitem 25? *

Copy and paste relevant sections from the manuscript (include quotes in quotation marks "like this" to indicate direct quotes from your manuscript), or elaborate on this item by providing additional information not in the ms, or briefly explain why the item is not applicable/relevant for your study

Like this: "...This study was funded by the U.S. National Institutes of Health, National Institute of Diabetes and Digestive and Kidney Diseases, under award #R34DK114773"

X27) Conflicts of Interest (not a CONSORT item)

X27-i) State the relation of the study team towards the system being evaluated

In addition to the usual declaration of interests (financial or otherwise), also state the relation of the study team towards the system being evaluated, i.e., state if the authors/evaluators are distinct from or identical with the developers/sponsors of the intervention.

	1	2	3	4	5	
subitem not at all important	○	○	○	◉	○	essential

Clear selection

Does your paper address subitem X27-i?

Copy and paste relevant sections from the manuscript (include quotes in quotation marks "like this" to indicate direct quotes from your manuscript), or elaborate on this item by providing additional information not in the ms, or briefly explain why the item is not applicable/relevant for your study

Like this: "...RTA received grant funding (R34DK114773) in support of this research and provides part-time clinical services as an affiliated physician of Northwestern Medicine. KAC, DTL, CA, SAH, AJC, and BH received salary support from grant R34DK114773. ND is employed by Northwestern Medicine as a primary care physician located at the participating primary care practice. KD is employed by Northwestern Medicine and serves as Vice President of Operations. AC is employed by Fitness Formula Clubs as a Registered Dietitian Coordinator. The authors disclose no further conflicts of interest."

About the CONSORT EHEALTH checklist

As a result of using this checklist, did you make changes in your manuscript? *

- ○ yes, major changes
- ◉ yes, minor changes
- ○ no

What were the most important changes you made as a result of using this checklist?

Changed adjectives to highlight important reporting dimensions. Restated the purpose of the study at the start of the Discussion.

How much time did you spend on going through the checklist INCLUDING making changes in your manuscript *

3 hours

Your answer must have a minimum of 25 characters.

As a result of using this checklist, do you think your manuscript has improved? *

- ◉ yes
- ○ no
- ○ Other:

Would you like to become involved in the CONSORT EHEALTH group?

This would involve for example becoming involved in participating in a workshop and writing an "Explanation and Elaboration" document

- ○ yes
- ◉ no
- ○ Other:

Clear selection

Any other comments or questions on CONSORT EHEALTH

Although I made some changes and the manuscript was improved, the process of copying and pasting text was extremely time consuming.

STOP - Save this form as PDF before you click submit

To generate a record that you filled in this form, we recommend to generate a PDF of this page (on a Mac, simply select "print" and then select "print as PDF") before you submit it.

When you submit your (revised) paper to JMIR, please upload the PDF as supplementary file.

Don't worry if some text in the textboxes is cut off, as we still have the complete information in our database. Thank you!

Final step: Click submit !

Click submit so we have your answers in our database!

Submit Clear form

Never submit passwords through Google Forms.

This content is neither created nor endorsed by Google. - Terms of Service - Privacy Policy

Does this form look suspicious? Report

Google Forms
